# Association Between the Cholesterol–High-Density Lipoprotein–Glucose Index and Urinary Incontinence: A Prospective Nationally Representative Cohort Study

**DOI:** 10.3390/healthcare14131984

**Published:** 2026-07-03

**Authors:** Tian Xia, Pingzhou Chen, Jinfeng Wu, Jiawen Wang, Deng Lin

**Affiliations:** 1 Department of Urology, Xuzhou Central Hospital, Xuzhou 221000, China; 2 Shengli Clinical Medical College of Fujian Medical University, Fujian Provincial Hospital, Fuzhou 350001, China; 3 Department of Urology, Fuzhou University Affiliated Provincial Hospital, Fuzhou 350001, China

**Keywords:** urinary incontinence, CHG index, older adults, cohort study, prediction model

## Abstract

**Objective:** This study examined whether the cholesterol–high-density lipoprotein–glucose (CHG) index was associated with subsequent urinary incontinence (UI) among middle-aged and older adults and evaluated its contribution to 4-year UI risk prediction. **Methods:** A total of 2059 participants without UI at baseline were included and followed from Wave 2 to Wave 7 in this prospective cohort study. The relationship between the CHG index and subsequent UI was examined using Cox proportional hazards models, supplemented by restricted cubic spline and subgroup analyses. A 4-year prediction model for UI was developed using LASSO and Cox regression. **Results:** UI occurred in 308 participants during follow-up, yielding an incidence of 14.96%. An independent inverse association was observed between CHG levels and the risk of subsequent UI. After full adjustment, each 1-standard deviation increase in CHG corresponded to an 18% lower risk of UI (HR of 0.82, 95% CI from 0.72 to 0.93). Relative to the lowest CHG tertile, the hazard ratios were 0.68 (95% CI from 0.51 to 0.90) for T2 and 0.63 (95% CI from 0.47 to 0.85) for T3. The relationship was predominantly linear, and no significant effect modification was detected in subgroup analyses. The 4-year prediction model showed moderate performance (AUC 0.663), while incorporation of CHG led to modest improvements in discrimination and reclassification. **Conclusions:** Among middle-aged and older adults, higher CHG levels were independently related to a reduced risk of UI. The CHG index may provide complementary information for early risk assessment in this population, but should not be regarded as a stand-alone predictor.

## 1. Introduction

Urinary incontinence (UI) represents a common but insufficiently recognized health problem in middle-aged and older adults [1]. Among individuals aged 65 years and above, its prevalence has been estimated at 30–60%, with women disproportionately affected [2,3]. UI is not merely a bothersome symptom; it is associated with impaired quality of life, reduced social engagement, psychological distress, depression, anxiety, falls, fractures, and functional decline [4,5]. As the population continues to age, UI is expected to impose a growing burden on individuals, caregivers, and healthcare systems, which is likely to increase, underscoring the need to identify potentially modifiable determinants.

Metabolic abnormalities have increasingly been recognized as contributing to the development of UI. Obesity, diabetes, and metabolic syndrome have all been linked to lower urinary tract dysfunction [6,7,8,9]. A plausible explanation is that insulin resistance, as a shared feature of metabolic disorders, may adversely affect bladder and urethral function through oxidative stress, chronic inflammation, microvascular injury, and autonomic dysfunction [10,11,12]. Despite these observations, the epidemiological evidence remains incomplete. Existing studies have largely examined single metabolic indicators, including body size, glucose, or lipid measures [13,14,15], and therefore may not adequately reflect overall metabolic dysregulation. Moreover, most available studies are cross-sectional, limiting inference regarding temporal sequence [16,17].

Composite metabolic indices may therefore offer a more comprehensive way to characterize metabolic risk relevant to UI. The triglyceride-glucose index and related derivatives have been widely adopted as surrogate markers of insulin resistance and have demonstrated predictive utility for several metabolic outcomes [18,19,20,21]. By integrating information from multiple metabolic domains, these indices may better capture the joint and cumulative effects of metabolic disturbances. The CHG index, a composite indicator integrating total cholesterol (TC), fasting blood glucose (FBG), and high-density lipoprotein cholesterol (HDL-C) [22,23]. Unlike single lipid or glucose markers, CHG combines information from both lipid and glycaemic domains and may therefore capture a broader pattern of glucose–lipid metabolic disturbance [22,23]. This combined profile is biologically relevant because dysregulated glucose and lipid metabolism may contribute to lower urinary tract dysfunction through insulin resistance, chronic low-grade inflammation, oxidative stress, microvascular injury, and autonomic nervous system impairment. Established insulin-resistance surrogate indices, such as the triglyceride–glucose index, have been widely used in metabolic and cardiovascular research. However, whether CHG provides comparable or additional information in relation to urinary incontinence remains unclear.

Accordingly, CHG was evaluated in relation to subsequent UI among participants who were free of UI at baseline, and the incremental value of CHG for 4-year UI risk prediction was also assessed. This study may provide further epidemiological evidence regarding the metabolic basis of UI and inform early risk stratification in older adults.

## 2. Methods

### 2.1. Study Framework, Participant Selection, and Ethical Considerations

This study analyzed secondary data obtained from the English Longitudinal Study of Ageing (ELSA), which is publicly available through the UK Data Service [24,25]. The current analysis was conducted using anonymized data and adhered to the principles of the Declaration of Helsinki. Therefore, ethical approval for this study was not required, as it relied on publicly available secondary data from the ELSA.

The analysis was based on participants from ELSA, a nationally representative prospective cohort with repeated measurements of demographic characteristics, lifestyle factors, chronic conditions, biomarkers, and health outcomes. Wave 2 was selected as the baseline for the present study because blood biomarker data required for CHG calculation were relatively complete at this wave. Participants were followed from Wave 2 through Wave 7 for the ascertainment of incident UI.

**Figure 1:**
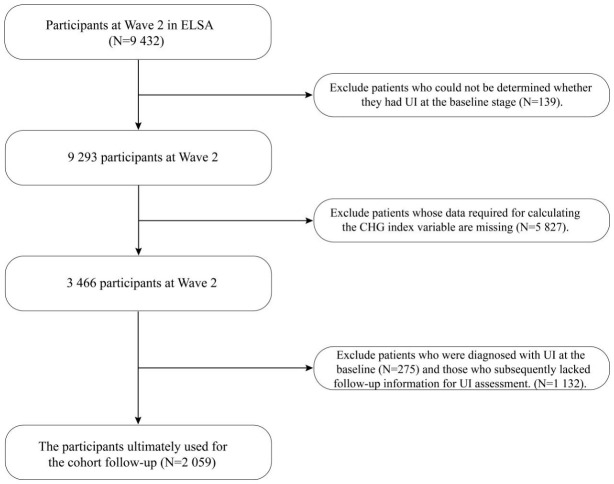
Flowchart of participant selection.

The study flow is presented in Figure 1. Among 9432 participants in Wave 2, 139 were excluded because baseline UI status was unclear. An additional 5827 participants were excluded because the data required for CHG calculation were missing. After further exclusion of 275 participants with baseline UI and 1132 without follow-up UI information, 2059 participants remained for the final longitudinal analysis.

### 2.2. Assessment of Exposure

Biochemical measurements were performed on fasting blood samples collected at baseline, including TC, HDL-C, and FBG. The CHG index was then calculated according to the following formula [22,23]:

$$CHG=ln\frac{TC\left(mg/dL\right)\;\times \;FBG(mg/dL)}{2\;\times HDL\text{-}C(mg/dL)}$$

For subsequent analyses, CHG was examined as a continuous variable and by tertiles, with effects also estimated per 1-standard deviation increase.

### 2.3. Outcome Ascertainment

UI was ascertained from self-reported items in the ELSA follow-up questionnaires. Participants were classified as having urinary incontinence if they reported urinary incontinence during the previous year and indicated that the condition had lasted for more than 1 month [26,27,28]. This questionnaire-based definition was applied consistently across follow-up waves. However, the available ELSA data did not provide sufficient information to distinguish stress urinary incontinence, urgency urinary incontinence, or mixed urinary incontinence. Therefore, the outcome was analyzed as overall incident urinary incontinence. Individuals with urinary incontinence at baseline were excluded, and those who first met this definition during follow-up were considered incident cases. Accordingly, the primary outcome was incident urinary incontinence occurring during follow-up.

### 2.4. Assessment of Covariates

To account for potential confounding, covariates were drawn from demographic, socioeconomic, lifestyle, and health-related domains [27,28]. Covariates covered demographic and socioeconomic characteristics, including age, sex, race, marital status, educational level, and income, as well as lifestyle-related factors such as smoking, alcohol use, and moderate-intensity physical activity. Baseline health status was described according to the presence of hypertension, diabetes, hypercholesterolemia, coronary heart disease, stroke, sleep disorders, self-rated health, body mass index (BMI), and C-reactive protein (CRP). BMI was calculated from measured height and weight at baseline, and CRP was treated as an inflammatory marker. TC, HDL-C, FBG, and CRP were obtained from baseline blood assays, whereas the remaining variables were mainly collected through questionnaires.

### 2.5. Statistical Analysis

Baseline characteristics were examined from two perspectives: by incident urinary incontinence during follow-up and by tertiles of CHG. Descriptive statistics were chosen according to variable distribution and data type. Continuous variables were presented as mean (standard deviation) or median (interquartile range), whereas categorical variables were shown as number (%). The statistical approach was determined by the nature and distribution of the variables. Two-group comparisons were conducted with the independent-samples *t* test or Mann–Whitney U test, tertile-based comparisons with one-way analysis of variance or the Kruskal–Wallis test, and categorical variables with the χ^2^ test.

Cox proportional hazards regression was used to evaluate the relationship between CHG and subsequent urinary incontinence, and the estimates are presented as hazard ratios with 95% confidence intervals. To provide a more complete assessment of the association, CHG was analyzed both continuously and by tertiles, with effect estimates additionally calculated for each 1-standard deviation increase. Cumulative incidence curves were used to visualize outcome occurrence across CHG categories, and trend analysis was performed by modeling the median value of each tertile as a continuous variable. Adjustment was performed in a stepwise manner: Model 1 included no covariates, Model 2 adjusted for demographic factors, and Model 3 additionally adjusted for clinically relevant variables. Prior to model construction, multicollinearity among covariates was examined using generalized variance inflation factors (GVIFs) (Supplementary Table S1), and the proportional hazards assumption was assessed on the basis of Schoenfeld residuals (Supplementary Figure S1 and Table S2).

The dose–response pattern between CHG and incident urinary incontinence was further explored with restricted cubic splines. To examine whether the tertile-based findings depended on the selected cut-points, additional sensitivity analyses were performed using alternative CHG categorizations, including tertile-combined groups, median split, quartile-based categories, and quartile-combined groups. These models used the same covariate adjustment strategy as the main Cox regression models. To assess the consistency of the association across population subgroups, stratified analyses were conducted according to sex, age, and other relevant variables, and interaction terms were used to test for between-subgroup heterogeneity.

For prediction modeling, candidate variables were first screened by LASSO regression and then entered into a Cox model to estimate 4-year urinary incontinence risk. The final model was visualized as a nomogram.

Its performance was examined in terms of discrimination, calibration, and clinical utility using the area under the receiver operating characteristic curve, Brier score, calibration plots, and decision curve analysis. Internal validation was performed through repeated 5-fold cross-validation. The incremental predictive role of CHG was quantified by the net reclassification improvement and integrated discrimination improvement.

Because a large number of participants were excluded owing to missing biomarker or follow-up data, baseline characteristics were compared between participants included in the final analytic sample and those excluded from the analysis. In addition, inverse probability weighting was used as a sensitivity analysis to examine the potential influence of differential inclusion. The probability of being included in the analytic sample was estimated using available baseline characteristics, and the resulting weights were incorporated into the Cox regression model. To compare CHG with established metabolic and insulin-resistance surrogate indices, additional analyses were conducted using TyG, AIP, TG/HDL-C, and METS-IR as alternative exposures. Each index was examined in relation to incident urinary incontinence using the same multivariable Cox regression framework. The predictive performance of models incorporating these indices was also compared to evaluate whether CHG provided information beyond commonly used metabolic indicators.

To assess whether early or potentially transient outcome events influenced the main association, a sensitivity analysis was conducted after excluding urinary incontinence events occurring within the first 2 years of follow-up. Additional sensitivity analyses were conducted to examine whether the association between CHG and incident urinary incontinence was influenced by frailty, functional status, medication use, and nutritional or physical performance indicators. The fully adjusted Cox model was further adjusted for frailty index, pain, mobility impairment and history of falls, antihypertensive medication use, diabetes medication use, hemoglobin, and gait speed. Sensitivity analyses were also performed after excluding participants with diabetes at baseline and after excluding participants with coronary heart disease or stroke at baseline. These analyses were used to assess whether the main association was materially affected by multidimensional vulnerability, functional impairment, medication use, anemia or nutritional reserve, sarcopenia-related physical performance, or major baseline cardiometabolic disease.

Analyses were carried out in RStudio (R version 4.5.1), and a two-sided *p* value < 0.05 indicated statistical significance. All figures were regenerated de novo from the analytic dataset using R. Survival curves were generated using the survival and survminer packages, restricted cubic spline plots and nomograms using the rms package, LASSO variable-selection plots using the glmnet package, and model-performance plots, including receiver operating characteristic curves, calibration plots, and decision curve analysis, using R-based graphical workflows.

## 3. Results

### 3.1. Participant Selection and Baseline Characteristics

After participant selection, 2059 individuals were retained for the longitudinal analysis. During follow-up, 308 participants developed incident UI, corresponding to an incidence of 14.96%. As summarized in Table 1, those who subsequently developed UI had lower baseline CHG levels, were older, had a higher BMI, and generally showed lower socioeconomic status, reflected by lower income and educational attainment. Women and unmarried individuals were also more common in this group. In addition, participants who developed UI were more likely to report poorer self-rated health, lower alcohol consumption, and less engagement in moderate-intensity physical activity, and they more frequently had sleep disorders and hypertension. Among the laboratory variables, only CRP differed between groups, whereas FBG, HbA1c, and lipid-related markers were comparable.

### 3.2. Baseline Profile According to CHG Tertiles

Baseline characteristics varied across CHG tertiles (Table 2). Higher CHG levels were accompanied by progressively higher values of BMI, FBG, HbA1c, TC, LDL, TG, and CRP, whereas HDL showed the opposite pattern. Those in the highest tertile had a higher prevalence of smoking, but were less likely to drink alcohol or undertake moderate physical activity. Hypertension and diabetes were more prevalent among those with higher CHG, while a history of stroke was more frequently observed in the lowest tertile. By contrast, income, marital status, race, self-rated health, sleep disorders, hypercholesterolemia, and coronary heart disease did not differ appreciably among tertiles. Although the proportion of incident UI declined numerically from 17.37% in T1 to 13.48% in T3, this difference was not statistically significant (*p* of 0.091).

### 3.3. Association of CHG with Incident UI

**Figure 2:**
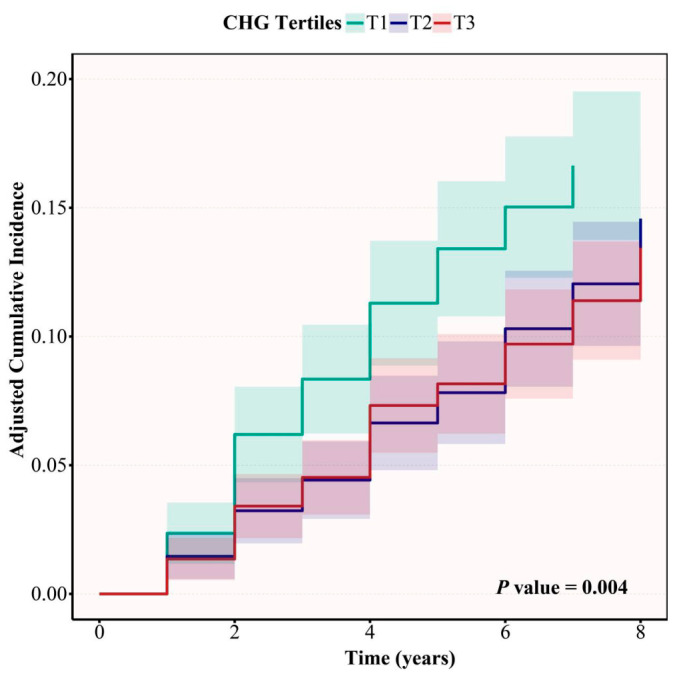
Adjusted cumulative incidence curves of incident urinary incontinence across CHG tertiles.

Figure 2 shows that participants in the lowest CHG tertile (T1) consistently exhibited the highest cumulative incidence of UI throughout follow-up, whereas the curves for T2 and T3 remained below that of T1. An overall group difference was detected among the three tertiles (*p* of 0.004).

Results from the Cox proportional hazards regression supported the survival analysis findings (Table 3). In the crude model, treating CHG as a continuous variable showed that each 1-unit increase in CHG corresponded to a 34% reduction in the risk of subsequent UI (HR of 0.66, 95% CI from 0.44 to 0.97, *p* of 0.037). This inverse association persisted after adjustment for demographic covariates (HR of 0.63, 95% CI from 0.42 to 0.94, *p* of 0.024), and became stronger after further adjustment for clinical confounders (HR of 0.49, 95% CI from 0.31 to 0.77, *p* of 0.002).

A similar pattern was observed when CHG was standardized. After full adjustment, each 1-standard deviation increase corresponded to an 18% reduction in the risk of subsequent UI (HR of 0.82, 95% CI from 0.72 to 0.93, *p* of 0.002).

When CHG was analyzed by tertiles, participants in T2 and T3 showed a lower risk of UI than those in T1. With T1 as the reference group, the fully adjusted HR were 0.68 (95% CI from 0.51 to 0.90) for T2 and 0.63 (95% CI from 0.47 to 0.85) for T3, with evidence of a linear trend across tertiles (*p* for trend of 0.002). Taken together, the findings consistently indicated an inverse association between CHG and incident UI, regardless of whether CHG was analyzed continuously or categorically. However, the absolute difference in crude UI incidence across CHG tertiles was modest, declining from 17.37% in T1 to 13.48% in T3, corresponding to an absolute difference of 3.89 percentage points.

### 3.4. Dose–Response Pattern and Subgroup Analyses

**Figure 3:**
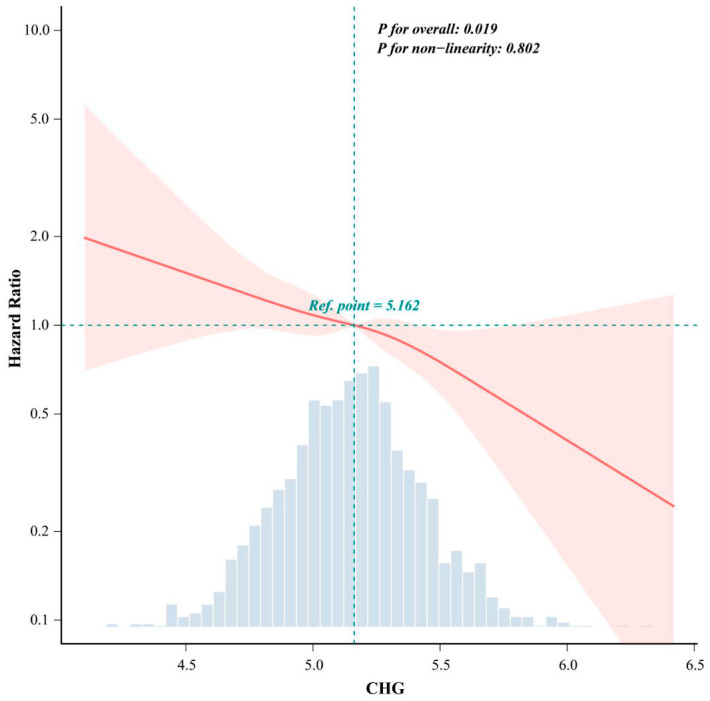
Restricted cubic spline analysis of the association between CHG and incident urinary incontinence.

RCS analysis indicated an overall association between CHG and incident UI (Figure 3, *p* for overall = 0.019), without evidence of nonlinearity (*p* for non-linearity = 0.802). These results support an approximately linear inverse relationship.

**Figure 4:**
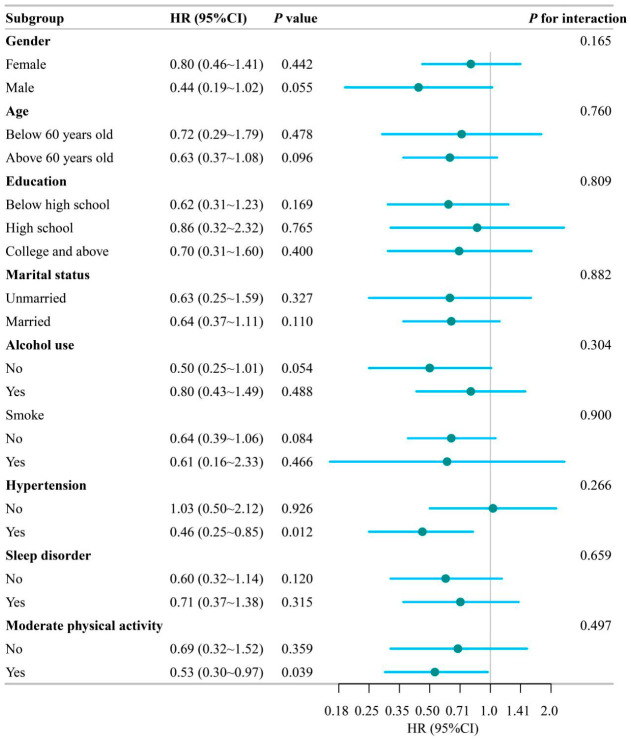
Subgroup analyses of the association between CHG and incident urinary incontinence.

Results of the subgroup analyses are presented in Figure 4. The inverse association between CHG and subsequent UI was generally similar across categories of sex, age, educational attainment, marital status, alcohol use, smoking, sleep disorders, hypertension, and physical activity. None of the interaction tests reached statistical significance (all *p* for interaction > 0.05). A more pronounced association was observed among participants with hypertension (HR of 0.46, 95% CI from 0.25 to 0.85, *p* of 0.012) and among those who engaged in moderate-intensity physical activity (HR of 0.53, 95% CI from 0.30 to 0.97, *p* of 0.039). Comparable trends were also seen in men, participants older than 60 years, and non-drinkers, although the corresponding confidence intervals included unity. Overall, there was no clear evidence of effect modification across subgroups.

### 3.5. Construction and Validation of a 4-Year UI Risk Prediction Model

#### 3.5.1. Variable Selection and Model Construction

**Figure 5:**
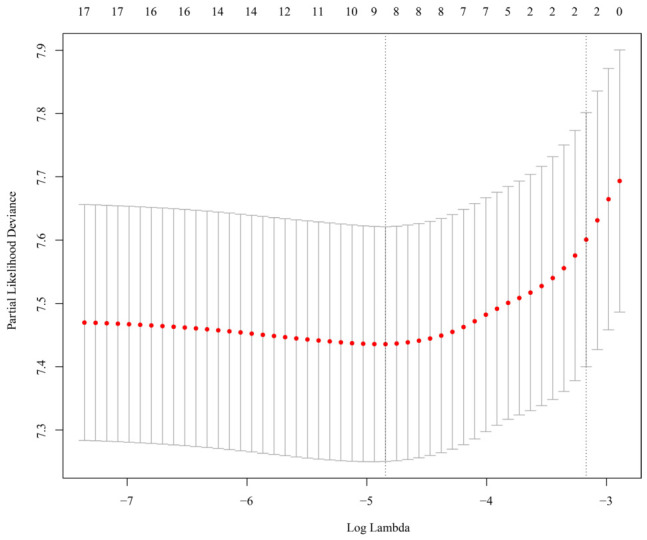
Cross-validation curve for LASSO variable selection.

Given the independent association between CHG and incident UI, its potential contribution to individualized risk prediction was further explored. As shown in Figure 5, the LASSO cross-validation curve was used to select the optimal penalty parameter for variable selection. The coefficient profiles of candidate predictors are presented in Supplementary Figure S2. At the optimal lambda, eight predictors remained in the model: CHG, BMI, age, educational level, marital status, sleep disorders, general health, and moderate physical activity. These variables were then incorporated into a nomogram to estimate 4-year UI risk (Supplementary Figure S3).

#### 3.5.2. Apparent Performance, Calibration, and Clinical Utility

**Figure 6:**
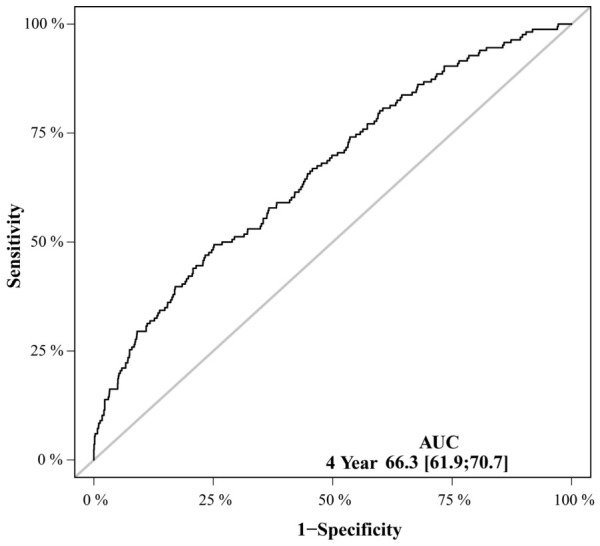
Receiver operating characteristic curve of the 4-year urinary incontinence prediction model.

Figure 6 and Table 4 summarize the discriminative performance of the model. The AUC and Brier score of the model were 0.663 and 0.072, respectively, indicating moderate discriminative capacity and acceptable overall performance. The calibration plot suggested close agreement between predicted and observed risk, without obvious systematic miscalibration (Supplementary Figure S4). According to the decision curve analysis, the model offered a higher net benefit than the treat-all and treat-none approaches over a threshold probability range of 5% to 93% (Supplementary Figure S5), indicating potential but limited clinical value. When net benefit was further examined at selected decision thresholds, adding CHG to the base model resulted in only small changes in net benefit. Across thresholds of 5% to 25%, the difference in net benefit between the CHG-added model and the base model ranged from −0.0011 to 0.0022 (Supplementary Table S15).

#### 3.5.3. Internal Validation

Internal validation results are shown in Table 4 and Supplementary Figure S6. Under 5-fold cross-validation, the model produced a mean AUC of 0.6466 and a mean Brier score of 0.0731. Compared with the apparent performance, the AUC decreased by 0.0164, whereas the Brier score increased by 0.0011. Repeated cross-validation gave a median AUC of 0.6483 (interquartile range from 0.604 to 0.681; range from 0.532 to 0.750) and a median Brier score of 0.0716 (interquartile range from 0.064 to 0.080; range from 0.058 to 0.105).

#### 3.5.4. Incremental Predictive Value of CHG

As shown in Table 5, incorporation of CHG into each of the three baseline models led to statistically significant but numerically modest improvements in both reclassification and discrimination. The NRIs for the three models were 0.119 (95% CI from 0.002 to 0.202; *p* of 0.032), 0.116 (95% CI from 0.001 to 0.190; *p* of 0.044), and 0.150 (95% CI from 0.016 to 0.205; *p* of 0.024), respectively. The corresponding IDIs were 0.005 (95% CI from 0.001 to 0.014; *p* < 0.001), 0.004 (95% CI from 0.001 to 0.011; *p* < 0.001), and 0.006 (95% CI from 0.002 to 0.015; *p* < 0.001).

Model 1 includes age, gender, education, marital status, race, and BMI. Model 2 includes alcohol use, smoking, diabetes, high cholesterol, hypertension, sleep disorder, moderate physical activity, job income, coronary heart disease, stroke, and general health. Model 3 includes age, gender, education, marital status, race, alcohol use, smoking, diabetes, high cholesterol, hypertension, sleep disorder, moderate physical activity, job income, coronary heart disease, stroke, general health, CRP, and BMI. Abbreviations: CHG, cholesterol, high-density lipoprotein, and glucose; UI, urinary incontinence; BMI, body mass index; CRP, C-reactive protein; IDI, integrated discrimination improvement; NRI, net reclassification improvement.

### 3.6. Sensitivity Analyses

#### 3.6.1. Assessment of Selection Bias Related to Complete-Case Inclusion

Baseline characteristics of included and excluded participants are shown in Supplementary Table S3. Compared with included participants, excluded participants were older and had lower income, higher BMI, higher CRP, higher HbA1c, higher triglyceride levels, slower gait speed, and higher frailty index values. Differences were also observed in several demographic, lifestyle, and clinical variables, including sex, education, smoking status, alcohol use, physical activity, hypertension, diabetes, coronary heart disease, stroke, and self-rated health.

In the inverse probability weighting sensitivity analysis, the association between CHG and incident urinary incontinence remained in the same direction as that observed in the complete-case analysis (Supplementary Table S4).

#### 3.6.2. Comparison with Established Metabolic Indices

The associations of CHG and other metabolic indices with incident urinary incontinence are shown in Supplementary Table S5. In the fully adjusted Cox models, CHG was associated with a lower risk of incident urinary incontinence per 1-SD increase (HR, 0.82; 95% CI, 0.72–0.93; *p* = 0.002). By contrast, TyG (HR, 0.90; 95% CI, 0.79–1.02; *p* = 0.100), TG/HDL-C ratio (HR, 0.90; 95% CI, 0.78–1.03; *p* = 0.131), AIP (HR, 0.89; 95% CI, 0.78–1.02; *p* = 0.097), CTI (HR, 0.95; 95% CI, 0.80–1.12; *p* = 0.543), SHR (HR, 0.96; 95% CI, 0.85–1.08; *p* = 0.478), METS-IR (HR, 0.78; 95% CI, 0.56–1.09; *p* = 0.143), and TyG-BMI (HR, 0.75; 95% CI, 0.50–1.14; *p* = 0.180) were not significantly associated with incident urinary incontinence.

#### 3.6.3. Sensitivity Analysis Excluding Early Urinary Incontinence Events

After excluding urinary incontinence events occurring within the first 2 years of follow-up, higher CHG remained associated with a lower risk of incident urinary incontinence in the fully adjusted model. The HR per 1-SD increase in CHG was 0.85 (95% CI, 0.73–0.98; *p* = 0.026). Compared with T1, the HRs were 0.78 (95% CI, 0.56–1.07; *p* = 0.123) for T2 and 0.68 (95% CI, 0.48–0.96; *p* = 0.030) for T3, with a significant trend across tertiles (*p* for trend = 0.028; Supplementary Table S6).

#### 3.6.4. Additional Adjustment and Exclusion-Based Sensitivity Analyses

Additional sensitivity analyses are shown in Supplementary Tables S7–S14. After further adjustment for frailty index, the inverse association between CHG and incident urinary incontinence remained evident. The HR per 1-SD increase in CHG was 0.81 (95% CI, 0.72–0.92; *p* < 0.001), and the HRs for T2 and T3 were 0.71 (95% CI, 0.54–0.93; *p* = 0.012) and 0.62 (95% CI, 0.47–0.84; *p* = 0.002), respectively (Supplementary Table S7). Similar results were observed after additional adjustment for pain, mobility impairment, and history of falls (Supplementary Table S8).

After additional adjustment for antihypertensive medication use, the HR per 1-SD increase in CHG was 0.81 (95% CI, 0.72–0.92; *p* < 0.001), and the HRs for T2 and T3 were 0.71 (95% CI, 0.54–0.93; *p* = 0.012) and 0.62 (95% CI, 0.46–0.83; *p* = 0.001), respectively (Supplementary Table S9). Similar estimates were observed after additional adjustment for diabetes medication use (Supplementary Table S10).

After additional adjustment for hemoglobin, the HR per 1-SD increase in CHG was 0.84 (95% CI, 0.74–0.95; *p* = 0.006), and the HRs for T2 and T3 were 0.72 (95% CI, 0.55–0.95; *p* = 0.019) and 0.67 (95% CI, 0.50–0.90; *p* = 0.009), respectively (Supplementary Table S11). After additional adjustment for gait speed, the HR per 1-SD increase in CHG was 0.82 (95% CI, 0.70–0.96; *p* = 0.014), and the HR for T3 remained statistically significant (HR, 0.59; 95% CI, 0.40–0.85; *p* = 0.005; Supplementary Table S12).

In exclusion-based sensitivity analyses, the inverse association remained after excluding participants with diabetes at baseline. The HR per 1-SD increase in CHG was 0.82 (95% CI, 0.72–0.92; *p* = 0.001), with HRs of 0.64 (95% CI, 0.48–0.85; *p* = 0.002) for T2 and 0.64 (95% CI, 0.48–0.86; *p* = 0.003) for T3 (Supplementary Table S13). Similar results were observed after excluding participants with coronary heart disease or stroke at baseline, with an HR per 1-SD increase of 0.83 (95% CI, 0.73–0.95; *p* = 0.005; Supplementary Table S14).

#### 3.6.5. Sensitivity Analyses Using Alternative CHG Cut-Points

To examine whether the tertile-based findings depended on the selected cut-points, additional analyses were conducted using alternative CHG categorizations (Supplementary Table S16). In the fully adjusted model, the inverse association remained evident when the two higher tertiles were combined and compared with the lowest tertile (T2–T3 vs. T1: HR, 0.66; 95% CI, 0.52–0.85; *p* = 0.001). A similar direction was observed using the median split (high vs. low: HR, 0.78; 95% CI, 0.62–1.00; *p* = 0.048). In quartile-based analyses, the HRs were 0.85 (95% CI, 0.62–1.17; *p* = 0.315) for Q2, 0.79 (95% CI, 0.57–1.10; *p* = 0.157) for Q3, and 0.64 (95% CI, 0.45–0.91; *p* = 0.013) for Q4, compared with Q1. The trend across quartiles was statistically significant (*p* for trend = 0.013). When Q2–Q4 were combined and compared with Q1, the association showed a similar direction but was borderline significant (HR, 0.77; 95% CI, 0.59–1.01; *p* = 0.056). These findings suggest that the categorical association was not solely driven by tertile-based classification, while the restricted cubic spline analysis did not support a clearly defined nonlinear threshold.

## 4. Discussion

The findings indicated that higher CHG levels were independently associated with a lower risk of subsequent UI in middle-aged and older adults. This inverse association remained stable when CHG was modeled continuously or by tertiles. RCS analysis suggested a largely linear relationship, and no significant heterogeneity was detected across subgroups. Although the tertile analysis suggested that participants in the lowest CHG group had the highest risk, the restricted cubic spline analysis did not support a clear nonlinear threshold. Additional analyses using alternative CHG cut-points showed a generally consistent direction of association, suggesting that the categorical findings were not solely driven by tertile-based classification. Therefore, CHG categories should be interpreted as exploratory risk-stratification groups rather than clinically established thresholds.

The direction of this association requires careful interpretation. Previous studies have linked obesity, diabetes, insulin resistance, and metabolic syndrome with lower urinary tract dysfunction [13,15,29]. If CHG were interpreted only as a conventional marker of adverse glucose–lipid metabolism, higher CHG would be expected to be associated with a higher, rather than lower, risk of UI. This was not observed in the present cohort. Therefore, the finding should not be interpreted as evidence that an unfavorable metabolic profile is protective. Instead, it suggests that the clinical meaning of CHG in middle-aged and older adults may extend beyond metabolic burden alone.

This interpretation is supported by the baseline profile of participants who developed UI. They were older and more likely to have poorer self-rated health, lower physical activity, sleep disorders, hypertension, and higher CRP. These features suggest that incident UI occurred in a group with greater baseline vulnerability, not simply in a group defined by glucose–lipid status. In later life, continence depends not only on bladder or urethral function, but also on the ability to recognize urinary urgency, move safely and quickly, maintain toileting routines, tolerate pain and sleep disturbance, and preserve daily functional independence [30,31,32]. UI in older adults should therefore be understood as a condition embedded in frailty, mobility limitation, falls, pain, sleep disturbance, medication burden, toileting difficulty, and declining physiological reserve [30,31,32,33]. More specifically, the cited continence and geriatric literature supports several complementary levels of interpretation. Evidence from frail and community-dwelling older adults indicates that UI is closely related to impaired mobility, disability, falls, pain, and reduced capacity to maintain toileting routines [30,31,32,33]. These domains overlap with the baseline profile observed in the present cohort and provide a clinical context in which lower CHG may coexist with poorer overall health, reduced reserve, and functional vulnerability. Therefore, the inverse CHG–UI association should not be interpreted as a purely metabolic signal, but as a finding that may partly reflect the interaction between biochemical status and multidimensional vulnerability in later life [30,31,32,33].

Recent work based on standardized nursing documentation helps place this issue in a broader clinical context. Cesare et al. analyzed nursing diagnoses using random forest modelling to identify patient profiles associated with ICU transfer risk and early clinical deterioration [34]. Although ICU transfer differs from UI, the study is relevant because it shows that standardized patient needs recorded through nursing diagnoses can capture clinically meaningful vulnerability and nursing complexity that may not be fully represented by laboratory values or medical diagnoses alone [34,35,36]. In adults, physical mobility impairment, injury risk, skin integrity impairment risk, acute pain, and fall risk were among the most informative nursing diagnoses [34]. This evidence is strengthened by previous nursing-informatics research showing that nursing diagnoses can be used as indicators of nursing complexity and that adding nursing data can improve the performance of predictive models for adverse hospital outcomes [34,35,36,37]. These domains are closely aligned with vulnerability indicators available in ELSA, including mobility impairment, pain, history of falls, frailty index, gait speed, hemoglobin, and sleep disturbance. Therefore, the additional sensitivity analyses in the present study should not be viewed merely as technical covariate adjustments. Rather, they provide an opportunity to examine whether the association between CHG and UI persists after accounting for measurable components of functional reserve, physical vulnerability, and nursing-sensitive patient needs.

After further adjustment for frailty index, pain, mobility impairment, history of falls, hemoglobin, and gait speed, the association between CHG and incident UI remained in the same direction. This finding does not prove a direct protective effect of CHG, nor does it indicate that metabolic status alone explains UI. Rather, CHG may provide routine biochemical information that is partly independent of these measured vulnerability domains, while still requiring interpretation alongside them. In older adults, lower CHG should not automatically be regarded as a healthier metabolic state. It may also coexist with reduced nutritional reserve, chronic disease burden, systemic inflammation, or functional decline that is not fully captured by conventional covariates [30,31,32,33]. Accordingly, the present findings should be interpreted as hypothesis-generating evidence that CHG may capture a glucose–lipid profile whose meaning differs across levels of physiological reserve and functional vulnerability. This interpretation is more consistent with the multidimensional nature of UI in older adults than with a simple organ-centered or metabolism-only explanation.

Several biological pathways may still contribute to the observed association. Insulin resistance may impair lower urinary tract control through autonomic and peripheral nerve injury, bladder sensory dysfunction, and detrusor or urethral sphincter abnormalities [38,39]. Chronic low-grade inflammation may also contribute to tissue injury, collagen remodeling, pelvic-floor muscle atrophy, and abnormal neural conduction [38,39,40]. However, these mechanisms should be considered part of a broader explanation rather than the whole interpretation. CHG is not a direct measure of insulin resistance. Unlike TyG and several triglyceride-based indices, it combines fasting glucose, total cholesterol, and HDL-C, and may therefore reflect a different glucose–lipid pattern. In older adults, this pattern may also be influenced by nutrition, comorbidity, inflammation, and functional reserve.

In the additional comparison with TyG, TG/HDL-C ratio, AIP, CTI, SHR, METS-IR, and TyG-BMI, CHG showed a statistically significant inverse association with incident UI, whereas the other examined indices did not reach statistical significance in the fully adjusted models. This finding suggests that CHG may capture a glucose–lipid pattern that is not fully represented by several commonly used insulin-resistance or lipid-related surrogate indices in this cohort. Nevertheless, this result should be interpreted cautiously. CHG should be considered a complementary marker of glucose–lipid status rather than a replacement for established insulin-resistance indices, and its incremental value requires further validation in independent populations.

Prediction tools for UI have mainly been developed in postoperative or postpartum settings, and models for community-dwelling older adults remain limited [41,42,43,44,45]. The predictive performance of the CHG-based model was broadly consistent with findings reported in previous studies [46]. In the present study, the CHG-based model showed acceptable calibration and stable internal validation, but its discriminative performance was only moderate, with an AUC of approximately 0.66. Therefore, the prediction results should be interpreted as exploratory rather than as evidence of immediate clinical applicability. Because the included predictors are routinely available, CHG may provide complementary information for risk assessment in community settings. However, the decision curve analysis at selected thresholds showed that the incremental net benefit of adding CHG to the base model was small. Therefore, CHG should be regarded as a complementary marker rather than a stand-alone predictor or replacement for established clinical factors. Importantly, the present model has only been internally validated within ELSA, and no external validation cohort was available in this study. External validation, particularly in non-UK cohorts with different demographic, healthcare, and risk-factor distributions, is needed before a CHG-based model can be considered for broader clinical decision-making.

A distinction should be made between statistical significance and clinical significance. In the association analyses, the fully adjusted HRs suggested a statistically significant inverse relationship between CHG and incident UI; however, the absolute difference in crude UI incidence between the lowest and highest CHG tertiles was 3.89 percentage points. This suggests that the association, although statistically robust, may correspond to a modest absolute risk difference at the population level. Similarly, the incremental prediction analyses showed statistically significant NRI and IDI values after adding CHG, but the magnitude of improvement was small. The decision curve analysis at selected thresholds also showed only limited changes in net benefit after adding CHG to the base model. Therefore, the present results support the epidemiological relevance of CHG as a complementary marker, but they do not establish CHG as a clinically actionable screening tool or a stand-alone decision-making variable. Its practical value would depend on whether future externally validated models demonstrate meaningful improvement in absolute risk classification, calibration, decision-curve benefit, and downstream clinical management.

The subgroup analyses should be interpreted cautiously. Although the point estimate appeared stronger among participants with hypertension, the interaction test was not statistically significant. This subgroup finding should therefore be interpreted as exploratory and may reflect differences in baseline risk, medication use, or statistical precision rather than definite effect modification. Additional adjustment for antihypertensive medication use yielded a similar direction of association, but residual confounding by specific antihypertensive drug classes, particularly diuretics, cannot be excluded.

The clinical implications of these findings should therefore be modest and context-dependent. CHG is easy to obtain from routine blood tests, but the present effect size and predictive performance do not support its use alone to identify older adults at risk of UI. A more clinically meaningful approach would be to interpret CHG together with urinary symptoms, mobility, falls, pain, sleep disturbance, frailty, nutritional reserve, medication use, comorbidity, and other nursing-sensitive indicators. This approach is consistent with how UI commonly presents in older adults: not only as a bladder-related symptom, but also as a sign of reduced reserve and increasing care needs. Future UI risk assessment may therefore be more useful if biochemical markers are combined with standardized assessment of patient needs and functional deterioration, rather than interpreted through a metabolic or organ-centered model alone. Before any clinical application, the incremental value of CHG should be confirmed in external cohorts and evaluated using absolute risk differences, calibration, decision-curve analysis, and clinically meaningful risk thresholds.

Several strengths should be acknowledged. The study’s longitudinal design provides stronger support for the temporal sequence underlying the observed association. Its large, nationally representative sample also increases the generalizability of the findings to community-dwelling older adults. In addition, the analyses accounted for a wide range of demographic, socioeconomic, lifestyle, and health-related covariates. The additional sensitivity analyses incorporating frailty, pain, mobility impairment, falls, hemoglobin, and gait speed further allowed us to examine whether the association was explained by several measurable domains of functional vulnerability and physiological reserve.

At the same time, several limitations should be considered. The large number of exclusions should be considered when interpreting the findings. Although ELSA is a nationally representative cohort, the present analysis was restricted to participants with available biomarker data, no urinary incontinence at baseline, and follow-up information. The comparison between included and excluded participants showed differences in several baseline characteristics, and inverse probability weighting was therefore performed as a sensitivity analysis. The direction of the main association was unchanged after weighting, but this approach cannot fully restore the representativeness of the original cohort. Further validation in independent cohorts with more complete biomarker and follow-up data is still needed. Urinary incontinence was identified by self-report rather than clinical assessment, and subtype-specific information was unavailable. Therefore, outcome misclassification may have occurred, and associations specific to stress or urgency urinary incontinence could not be examined. The sensitivity analysis excluding early urinary incontinence events showed a similar direction of association, but clinically validated and subtype-specific outcome assessment is still needed in future studies. Residual confounding may still be present because several important factors, including obstetric history, pelvic floor surgery, parity, number of vaginal births, menopausal status, diuretic use, depressive symptoms, and more detailed behavioral characteristics, were unavailable. Although additional sensitivity analyses adjusted for frailty index, pain, mobility impairment, history of falls, antihypertensive medication use, diabetes medication use, hemoglobin, and gait speed, these variables cannot fully substitute for unavailable obstetric, gynecological, medication-specific, or psychological covariates. In addition, although several ELSA variables reflected functional vulnerability and physiological reserve, including frailty, mobility impairment, pain, falls, gait speed, hemoglobin, and sleep disturbance, the dataset did not include standardized nursing diagnoses or direct measures of nursing complexity. Therefore, the nursing-sensitive interpretation proposed in this study is based on conceptually related indicators rather than formal nursing diagnostic data. Future studies linking biochemical markers with standardized nursing documentation and longitudinal functional assessment may better clarify how metabolic status, patient needs, and nursing complexity jointly contribute to UI risk in older adults. Finally, although the prediction model showed acceptable calibration and stable internal validation, its discriminative performance was only moderate, and the incremental net benefit of adding CHG was small at selected decision thresholds. CHG should therefore be regarded as a complementary rather than stand-alone marker. Because the model was developed and internally validated using ELSA data, its transportability to non-UK populations remains uncertain. Future studies should externally validate and recalibrate the model in independent cohorts from different countries before clinical implementation.

## 5. Conclusions

Higher CHG levels were associated with a lower risk of incident urinary incontinence in this longitudinal ELSA analysis. CHG may provide complementary information for risk assessment, but the moderate discriminative performance and limited incremental net benefit of the prediction model indicate that it should not be regarded as a stand-alone predictor. Further studies with external validation in non-UK populations, model recalibration when necessary, and more detailed urinary incontinence assessment are needed before broader clinical application.

## Figures and Tables

**Table 1:** Baseline Characteristics of Participants According to Incident UI in the Longitudinal Cohort.

Variable	Overall (N = 2059)	Non-UI (N = 1751)	UI (N = 308)	*p*-Value
CHG	5.16 ± 0.28	5.16 ± 0.29	5.12 ± 0.27	**0.037**
Demographic characteristics				
Age (year)	62.96 ± 7.09	62.49 ± 6.88	65.66 ± 7.68	**<0.001**
BMI (kg/m^2^)	27.57 ± 4.55	27.44 ± 4.44	28.30 ± 5.03	**0.002**
Income	8877.83 (4057.64, 14,590.45)	8982.91 (4157.60, 14,825.72)	7842.09 (3848.00, 12,514.14)	**0.032**
Gender				**<0.001**
Female	1107 (53.76)	894 (51.06)	213 (69.16)	
Male	952 (46.24)	857 (48.94)	95 (30.84)	
Marital status				**0.002**
Unmarried	503 (24.43)	406 (23.19)	97 (31.49)	
Married	1556 (75.57)	1345 (76.81)	211 (68.51)	
Education				**<0.001**
Below high school	675 (32.78)	543 (31.01)	132 (42.86)	
High school	593 (28.80)	516 (29.47)	77 (25.00)	
College and above	791 (38.42)	692 (39.52)	99 (32.14)	
Race				0.157
Non-white	24 (1.17)	18 (1.03)	6 (1.95)	
White	2035 (98.83)	1733 (98.97)	302 (98.05)	
General health				**<0.001**
Excellent	372 (18.07)	337 (19.25)	35 (11.36)	
Very good	718 (34.87)	631 (36.04)	87 (28.25)	
Good	643 (31.23)	547 (31.24)	96 (31.17)	
Fair	277 (13.45)	205 (11.71)	72 (23.38)	
Poor	49 (2.38)	31 (1.77)	18 (5.84)	
Lifestyle factors				
Smoke				0.239
No	1803 (87.57)	1527 (87.21)	276 (89.61)	
Yes	256 (12.43)	224 (12.79)	32 (10.39)	
Drink				**0.014**
No	734 (35.65)	607 (34.67)	127 (41.23)	
Yes	1325 (64.35)	1144 (65.33)	181 (58.77)	
Moderate physical activity				**<0.001**
No	589 (28.61)	474 (27.07)	115 (37.34)	
Yes	1470 (71.39)	1277 (72.93)	193 (62.66)	
Health status				
Sleep disorder				**<0.001**
No	1256 (61.00)	1096 (62.59)	160 (51.95)	
Yes	803 (39.00)	655 (37.41)	148 (48.05)	
Hypertension				**0.035**
No	1030 (50.02)	893 (51.00)	137 (44.48)	
Yes	1029 (49.98)	858 (49.00)	171 (55.52)	
Diabetes				0.970
No	1978 (96.07)	1682 (96.06)	296 (96.1)	
Yes	81 (3.93)	69 (3.94)	12 (3.9)	
High cholesterol				0.499
No	1712 (83.15)	1460 (83.38)	252 (81.82)	
Yes	347 (16.85)	291 (16.62)	56 (18.18)	
Coronary heart disease				0.070
No	1938 (94.12)	1655 (94.52)	283 (91.88)	
Yes	121 (5.88)	96 (5.48)	25 (8.12)	
Stroke				0.072
No	2019 (98.06)	1721 (98.29)	298 (96.75)	
Yes	40 (1.94)	30 (1.71)	10 (3.25)	
Laboratory variables				
CRP (mg/L)	1.80 (0.80, 3.70)	1.70 (0.80, 3.50)	2.30 (1.00, 4.70)	**<0.001**
FBG (mg/dL)	89.58 ± 14.02	89.66 ± 14.34	89.14 ± 12.04	0.547
HbA1C	5.46 ± 0.47	5.46 ± 0.48	5.47 ± 0.40	0.610
TC (mg/dL)	234.93 ± 44.92	235.21 ± 44.98	233.39 ± 44.62	0.513
HDL-C (mg/dL)	60.71 ± 14.81	60.49 ± 14.78	61.95 ± 14.91	0.112
LDL-C (mg/dL)	146.53 ± 37.42	146.84 ± 37.32	144.80 ± 38.00	0.379
TG (mg/dL)	139.86 ± 79.30	140.90 ± 81.50	133.95 ± 65.19	0.156

Values are n (%) or mean  ±  SD or median (quartile). Abbreviation: CHG, cholesterol, high-density lipoprotein, and glucose; UI, urinary incontinence; BMI, body mass index; CRP, C-reactive protein; HbA1c, glycosylated hemoglobin; LDL-C, low-density lipoprotein cholesterol; HDL-C, high-density lipoprotein cholesterol; TC, total cholesterol; FBG, fasting blood glucose. *p*-value less than 0.05 is expressed in bold.

**Table 2:** Baseline Characteristics of Participants Classified According to the Tertiles of CHG.

Variable	Overall (N = 2059)	CHG T1 (N = 685)	CHG T2 (N = 684)	CHG T3 (N = 690)	*p*-Value
Demographic characteristics					
Age (year)	62.96 ± 7.09	62.43 ± 7.15	63.49 ± 7.24	62.98 ± 6.86	**0.023**
BMI (kg/m^2^)	27.57 ± 4.55	26.10 ± 4.13	27.38 ± 4.21	29.21 ± 4.73	**<0.001**
Income	8877.83 (4057.64, 14,590.45)	9100.00 (3602.77, 14,663.16)	8320.41 (4209.81, 14,049.85)	9227.10 (4294.47, 14,766.27)	0.223
Gender					**<0.001**
Female	1107 (53.76)	444 (64.82)	377 (55.12)	286 (41.45)	
Male	952 (46.24)	241 (35.18)	307 (44.88)	404 (58.55)	
Marital status					0.495
Unmarried	503 (24.43)	175 (25.55)	170 (24.85)	158 (22.9)	
Married	1556 (75.57)	510 (74.45)	514 (75.15)	532 (77.1)	
Education					**0.036**
Below high school	675 (32.78)	193 (28.18)	242 (35.38)	240 (34.78)	
High school	593 (28.80)	216 (31.53)	187 (27.34)	190 (27.54)	
College and above	791 (38.42)	276 (40.29)	255 (37.28)	260 (37.68)	
Race					0.875
Non-white	24 (1.17)	9 (1.31)	8 (1.17)	7 (1.01)	
White	2035 (98.83)	676 (98.69)	676 (98.83)	683 (98.99)	
General health					0.579
Excellent	372 (18.07)	140 (20.44)	118 (17.25)	114 (16.52)	
Very good	718 (34.87)	233 (34.01)	249 (36.4)	236 (34.2)	
Good	643 (31.23)	202 (29.49)	218 (31.87)	223 (32.32)	
Fair	277 (13.45)	92 (13.43)	85 (12.43)	100 (14.49)	
Poor	49 (2.38)	18 (2.63)	14 (2.05)	17 (2.46)	
Lifestyle factors					
Smoke					**0.017**
No	1803 (87.57)	609 (88.91)	610 (89.18)	584 (84.64)	
Yes	256 (12.43)	76 (11.09)	74 (10.82)	106 (15.36)	
Drink					**0.014**
No	734 (35.65)	223 (32.55)	236 (34.5)	275 (39.86)	
Yes	1325 (64.35)	462 (67.45)	448 (65.5)	415 (60.14)	
Moderate physical activity					**<0.001**
No	589 (28.61)	168 (24.53)	182 (26.61)	239 (34.64)	
Yes	1470 (71.39)	517 (75.47)	502 (73.39)	451 (65.36)	
Health status					
Sleep disorder					0.932
No	1256 (61.00)	415 (60.58)	421 (61.55)	420 (60.87)	
Yes	803 (39.00)	270 (39.42)	263 (38.45)	270 (39.13)	
Hypertension					**<0.001**
No	1030 (50.02)	379 (55.33)	346 (50.58)	305 (44.2)	
Yes	1029 (49.98)	306 (44.67)	338 (49.42)	385 (55.8)	
Diabetes					**<0.001**
No	1978 (96.07)	677 (98.83)	673 (98.39)	628 (91.01)	
Yes	81 (3.93)	8 (1.17)	11 (1.61)	62 (8.99)	
High cholesterol					0.511
No	1712 (83.15)	565 (82.48)	578 (84.5)	569 (82.46)	
Yes	347 (16.85)	120 (17.52)	106 (15.5)	121 (17.54)	
Coronary heart disease					0.151
No	1938 (94.12)	635 (92.7)	648 (94.74)	655 (94.93)	
Yes	121 (5.88)	50 (7.3)	36 (5.26)	35 (5.07)	
stroke					**0.004**
No	2019 (98.06)	663 (96.79)	671 (98.10)	685 (99.28)	
Yes	40 (1.94)	22 (3.21)	13 (1.90)	5 (0.72)	
Incidence of UI					0.091
No	1751 (85.04)	566 (82.63)	588 (85.96)	597 (86.52)	
Yes	308 (14.96)	119 (17.37)	96 (14.04)	93 (13.48)	
Laboratory variables					
CRP (mg/L)	1.80 (0.80, 3.70)	1.40 (0.60, 2.80)	1.70 (0.90, 3.30)	2.30 (1.20, 4.80)	**<0.001**
FBG (mg/dL)	89.58 ± 14.02	83.75 ± 8.37	87.91 ± 8.45	97.04 ± 18.83	**<0.001**
HbA1C	5.46 ± 0.47	5.35 ± 0.36	5.42 ± 0.34	5.60 ± 0.62	**<0.001**
TC (mg/dL)	234.93 ± 44.92	219.51 ± 41.75	236.60 ± 42.94	248.59 ± 45.20	**<0.001**
HDL-C (mg/dL)	60.71 ± 14.81	71.77 ± 14.62	59.75 ± 11.24	50.68 ± 9.70	**<0.001**
LDL-C (md/dL)	146.53 ± 37.42	128.25 ± 32.08	150.83 ± 34.11	160.41 ± 38.27	**<0.001**
TG (mg/dL)	139.86 ± 79.30	98.47 ± 42.45	131.15 ± 54.58	189.58 ± 98.94	**<0.001**

Values are n (%) or mean  ±  SD or median (quartile). Abbreviation: CHG, cholesterol, high-density lipoprotein, and glucose; UI, urinary incontinence; BMI, body mass index; CRP, C-reactive protein; HbA1c, glycosylated hemoglobin; LDL-C, low-density lipoprotein cholesterol; HDL-C, high-density lipoprotein cholesterol; TC, total cholesterol; FBG, fasting blood glucose. *p*-value less than 0.05 is expressed in bold.

**Table 3:** Association between the CHG and UI incidence in patients.

Variables	Model 1		Model 2		Model 3	
	HR (95% CI)	*p* Value	HR (95% CI)	*p* Value	HR (95% CI)	*p* Value
CHG (per 1 unit)	0.66 (0.44~0.97)	0.037	0.63 (0.42~0.94)	0.024	0.49 (0.31~0.77)	0.002
CHG (per 1 SD)	0.89 (0.79~0.99)	0.037	0.88 (0.78~0.98)	0.024	0.82 (0.72~0.93)	0.002
CHG Tertiles						
T1	1.0 [Ref]		1.0 [Ref]		1.0 [Ref]	
T2	0.79 (0.61~1.04)	0.090	0.73 (0.56~0.96)	0.022	0.68 (0.51~0.90)	0.007
T3	0.76 (0.58~0.99)	0.044	0.73 (0.56~0.96)	0.026	0.63 (0.47~0.85)	0.002
*p*-trend	0.87 (0.76~0.99)	0.041	0.85 (0.74~0.98)	0.023	0.79 (0.68~0.92)	0.002

Model 1: unadjusted for any covariates. Model 2: adjusted for age, gender, education, marital status, and race. Model 3: adjusted for age, gender, education, marital status, race, alcohol use, smoking, diabetes, high cholesterol, hypertension, sleep disorder, moderate physical activity, job income, coronary heart disease, stroke, general health, CRP, and BMI. Abbreviation: CHG, cholesterol, high-density lipoprotein, and glucose; UI, urinary incontinence; BMI, body mass index; CRP, C-reactive protein; HR, hazard ratio.

**Table 4:** Internal validation performance of the Cox model.

Section	Statistic	AUC	Brier Score
Model performance	Apparent performance	0.663	0.072
	Internal CV mean	0.647	0.073
	Change after validation	−0.016	0.001
Repeated CV	Median	0.648	0.072
	IQR (Q1–Q3)	0.604–0.681	0.064–0.080
	Range (min–max)	0.532–0.750	0.058–0.105
	SD	0.051	0.010

Notes: Higher AUC indicates better discrimination, whereas lower Brier score indicates better overall predictive accuracy. Internal validation used 5-fold cross-validation with 100 partitions and 50 repeated results. Overall interpretation: moderate discrimination with relatively stable internal validation performance.

**Table 5:** Improvement in Risk Prediction After Adding CHG to three Models.

Event	NRI (95%CI)	NRI *p*-Value	IDI (95%CI)	IDI *p*-Value
Model 1	Ref		Ref	
Model 1 + CHG	0.119 (0.002–0.202)	0.032	0.005 (0.001–0.014)	<0.001
Model 2	Ref		Ref	
Model 2 + CHG	0.116 (0.001–0.190)	0.044	0.004 (0.001–0.011)	<0.001
Model 3	Ref		Ref	
Model 3 + CHG	0.150 (0.016–0.205)	0.024	0.006 (0.002–0.015)	<0.001

## Data Availability

The data used in this study are available from the English Longitudinal Study of Ageing (ELSA) through the UK Data Service, subject to registration and the relevant access conditions.

## Supplementary Materials

The following supporting information can be downloaded at: https://www.mdpi.com/article/10.3390/healthcare14131984/s1, Figure S1. Assessment of the proportional hazards assumption for CHG. Figure S2. Coefficient profiles of candidate predictors in the LASSO regression. Figure S3. Nomogram for estimating 4-year urinary incontinence risk. Figure S4. Calibration plot of the 4-year urinary incontinence prediction model. Figure S5. Decision curve analysis of the 4-year urinary incontinence prediction model. Figure S6. Internal validation performance of the 4-year urinary incontinence prediction model. Table S1. Multicollinearity diagnostics for covariates included in the fully adjusted CHG model. Table S2. Tests of the Proportional Hazards Assumption Based on Schoenfeld Residuals. Table S3. Baseline characteristics of included and excluded participants. Table S4. Sensitivity analysis using inverse probability weighting for the association between CHG and incident urinary incontinence. Table S5. Comparison of CHG with established metabolic and insulin-resistance surrogate indices. Table S6. Sensitivity analysis excluding urinary incontinence events within the first 2 years. Table S7. Sensitivity analysis of the association between CHG and incident urinary incontinence after additional adjustment for frailty index. Table S8. Sensitivity analysis of the association between CHG and incident urinary incontinence after additional adjustment for pain, mobility impairment, and history of falls. Table S9. Sensitivity analysis of the association between CHG and incident urinary incontinence after additional adjustment for antihypertensive medication use. Table S10. Sensitivity analysis of the association between CHG and incident urinary incontinence after additional adjustment for diabetes medication use. Table S11. Sensitivity analysis of the association between CHG and incident urinary incontinence after additional adjustment for hemoglobin. Table S12. Sensitivity analysis of the association between CHG and incident urinary incontinence after additional adjustment for gait speed. Table S13. Sensitivity analysis excluding participants with diabetes at baseline. Table S14. Sensitivity analysis excluding participants with coronary heart disease or stroke at baseline. Table S15. Net benefit of the base model and the CHG-added model at selected decision thresholds. Table S16. Sensitivity analyses using alternative CHG cut-points for incident urinary incontinence.

## Abbreviations

AUC	area under the receiver operating characteristic curve
BMI	body mass index
CHG	cholesterol–high-density lipoprotein–glucose
CI	confidence interval
CRP	C-reactive protein
DCA	decision curve analysis
ELSA	English Longitudinal Study of Ageing
FBG	fasting blood glucose
GVIF	generalized variance inflation factor
HbA1c	glycated hemoglobin
HDL-C	high-density lipoprotein cholesterol
HR	hazard ratio
IDI	integrated discrimination improvement
LASSO	least absolute shrinkage and selection operator
LDL	low-density lipoprotein cholesterol
NRI	net reclassification improvement
RCS	restricted cubic spline
SD	standard deviation
TC	total cholesterol
TG	triglycerides
UI	urinary incontinence
