# Supplementary material for: Association Between the Cholesterol–High-Density Lipoprotein–Glucose Index and Urinary Incontinence: A Prospective Nationally Representative Cohort Study
Source: Healthcare (Basel). 2026 Jul 3;14(13):1984. doi: 10.3390/healthcare14131984 (PMC13362180; doi:10.3390/healthcare14131984)
Supplement: Supplementary file 1 [file healthcare-14-01984-s001.zip › healthcare-4339575-supplementary.pdf]

Supplementary Table S1. Multicollinearity diagnostics for covariates included in the fully adjusted CHG model.

Variables	GVIF	DF	GVIF^(1/(2*Df))	colinearity (0=no, 1=yes)
CHG	1.214	1	1.102	0
CRP	1.104	1	1.051	0
BMI	1.287	1	1.134	0
age	1.353	1	1.163	0
education	1.159	2	1.038	0
marital	1.069	1	1.034	0
race	1.041	1	1.020	0
drink	1.091	1	1.044	0
smoke	1.081	1	1.040	0
hypertension	1.143	1	1.069	0
high cholesterol	1.061	1	1.030	0
diabetes	1.107	1	1.052	0
sleepdisorder	1.085	1	1.042	0
moderatePA	1.129	1	1.062	0
jobincome	1.228	1	1.108	0
CHD	1.098	1	1.048	0
stroke	1.089	1	1.044	0
general health	1.353	4	1.039	0

Abbreviations: CHG, cholesterol, high-density lipoprotein, and glucose; UI, urinary incontinence; BMI, Body mass index; GVIF, Generalized variance inflation factor; DF, degree of freedom; CHD, coronary heart disease; HBA1c, hemoglobin Alc.

Supplementary Table S2. Tests of the Proportional Hazards Assumption Based on Schoenfeld Residuals.

Variable	chisq	df	p.value
CRP	0.123	1	0.725
BMI	0.556	1	0.456
age	4.496	1	0.034
gender	0.611	1	0.434
education	0.004	1	0.947
marital	0.184	1	0.668
ethnic	0.014	1	0.905
drinkpastyear	3.886	1	0.049
smoke	0.857	1	0.355
hypertension	0.016	1	0.901
diabetes	0.174	1	0.677
sleepdisorder	0.013	1	0.910
moderatePA	0.077	1	0.781
jobincome	10.754	1	0.001
CHD	1.190	1	0.275
stroke	0.970	1	0.325
general_health	25.221	1	0.000
high_cholesterol.data	0.935	1	0.334
CHG	0.283	1	0.595
GLOBAL	31.571	22	0.085

Abbreviations: CHG, cholesterol, high-density lipoprotein, and glucose; UI, urinary incontinence; BMI, Body mass index; CHD, coronary heart disease; HBA1c, hemoglobin Alc.

Table S3. Baseline characteristics of included and excluded participants.

Variable	Included participants (N = 2,059)	Excluded participants (N = 7,373)	*P*-value
**CHG**	5.16 ± 0.28	5.18 ± 0.29	***0.045***
**Demographic characteristics**			
**Age (year)**	62.96 ± 7.09	66.45 ± 11.12	***<0.001***
**BMI (kg/m²)**	27.57 ± 4.56	28.07 ± 5.01	***<0.001***
**Income**	8877.83 (4057.64, 14590.45)	7721.94 (4212.00, 12624.20)	***<0.001***
**Gender**			***0.010***
Female	1107 (53.76)	4200 (56.96)	
Male	952 (46.24)	3173 (43.04)	
**Marital status**			***<0.001***
Unmarried	503 (24.43)	2664 (36.13)	
Married	1556 (75.57)	4709 (63.87)	
**Education**			***<0.001***
Below high school	675 (32.78)	3075 (41.71)	
High school	593 (28.80)	1516 (20.56)	
College and above	791 (38.42)	2782 (37.73)	
**Race**			***<0.001***
Non-white	24 (1.17)	216 (2.93)	
White	2035 (98.83)	7157 (97.07)	
**Lifestyle factors**			
**Smoke**			***<0.001***
No	1803 (87.57)	6080 (82.46)	
Yes	256 (12.43)	1293 (17.54)	
**Drink**			***<0.001***
No	734 (35.65)	1943 (26.35)	
Yes	1325 (64.35)	5430 (73.65)	
**Moderate physical activity**			***<0.001***
No	589 (28.61)	1635 (22.18)	
Yes	1470 (71.39)	5738 (77.82)	
**Health status**			
**Sleep disorder**			***0.022***
No	1256 (61.00)	4291 (58.20)	
Yes	803 (39.00)	3082 (41.80)	
**Hypertension**			***<0.001***
No	1030 (50.02)	3383 (45.88)	
Yes	1029 (49.98)	3990 (54.12)	
**Diabets**			***<0.001***
No	1978 (96.07)	6568 (89.08)	
Yes	81 (3.93)	805 (10.92)	
**High cholesterol**			0.630

No	1712 (83.15)	6097 (82.69)	
Yes	347 (16.85)	1276 (17.31)	
**Coronary heart disease**			***<0.001***
No	1938 (94.12)	6348 (86.10)	
Yes	121 (5.88)	1025 (13.90)	
**Stroke**			***<0.001***
No	2019 (98.06)	6969 (94.52)	
Yes	40 (1.94)	404 (5.48)	
**Pain**			***<0.001***
No	1428 (69.35)	4491 (60.91)	
Yes	631 (30.65)	2882 (39.09)	
**Mobility impairment**			***<0.001***
No	1948 (94.61)	6307 (85.54)	
Yes	111 (5.39)	1066 (14.46)	
**History of falls**			***<0.001***
No	1692 (82.18)	5718 (77.55)	
Yes	367 (17.82)	1655 (22.45)	
**Frailty index**			***<0.001***
No	1565 (76.01)	5304 (71.94)	
Yes	494 (23.99)	2069 (28.06)	
**Laboratory variables**			
**CRP (mg/L)**	1.80 (0.80, 3.70)	2.20 (1.00, 4.50)	***<0.001***
**FBG (mg/dL)**	89.58 ± 14.02	91.23 ± 20.04	***0.007***
**HbA1C**	5.46 ± 0.47	5.68 ± 0.82	***<0.001***
**TC (mg/dL)**	234.93 ± 44.92	225.19 ± 47.10	***<0.001***
**HDL (mg/dL)**	60.71 ± 14.81	57.98 ± 15.05	***<0.001***
**LDL (mg/dL)**	146.79 ± 37.25	134.26 ± 38.67	***<0.001***
**TG (mg/dL)**	139.86 ± 79.30	140.90 ± 81.50	0.156
**Hemoglobin (g/L)**	145.51 ± 12.99	142.32 ± 14.29	***<0.001***

Values are n (%) or mean ± SD or median (quartile).

Abbreviation: CHG, cholesterol, high-density lipoprotein, and glucose; UI, urinary incontinence; BMI, Body mass index; CRP, C-reactive protein; HbA1c, glycosylated hemoglobin; LDL-C, Low-density lipid cholesterol; HDL-C, High-density lipoprotein cholesterol; TC, Total cholesterol; FBG, Fasting blood glucose.

P - value less than 0.05 is expressed in bold.

Table S4. Sensitivity analysis using inverse probability weighting for the association between CHG and incident urinary incontinence

Analysis	CHG specification	N	Events	HR (95% CI)	P value
Complete-case Cox model	Per 1-unit increase	2009	298	0.64 (0.40–1.01)	0.056
	Per 1-SD increase	2009	298	0.88 (0.77–1.00)	0.056
	Tertile 1	2009	298	1.00 [Reference]	—
	Tertile 2	2009	298	0.74 (0.56–0.98)	0.035
	Tertile 3	2009	298	0.75 (0.55–1.02)	0.064
	P for trend	2009	298	0.56 (0.31–0.99)	0.048
IPW-weighted Cox model	Per 1-SD increase	2009	298	0.90 (0.79–1.01)	0.080
	Tertile 1	2009	298	1.00 [Reference]	—
	Tertile 2	2009	298	0.72 (0.55–0.94)	0.015
	Tertile 3	2009	298	0.85 (0.63–1.14)	0.268
	P for trend	2009	298	0.67 (0.39–1.17)	0.161

Values are hazard ratios and 95% confidence intervals estimated using Cox proportional hazards regression.
The complete-case model was adjusted for age, sex, education, marital status, race, alcohol use, smoking, diabetes, high cholesterol, hypertension, sleep disorder, moderate physical activity, income, coronary heart disease, stroke, general health, C-reactive protein, and body mass index.
The IPW-weighted model used stabilized inverse probability weights estimated from available baseline characteristics; weights were truncated at the 1st and 99th percentiles.
P for trend was calculated by modeling the median value of each CHG tertile as a continuous variable.
Abbreviations: CHG, cholesterol–high-density lipoprotein–glucose index; CI, confidence interval; HR, hazard ratio; IPW, inverse probability weighting; UI, urinary incontinence.

Table S5. Comparison of CHG with established metabolic and insulin-resistance surrogate indices

Index	HR per 1 SD (95% CI)	P value
CHG	0.82 (0.72~0.93)	0.002
TyG	0.90 (0.79–1.02)	0.100
TG/HDL-C ratio	0.90 (0.78–1.03)	0.131
AIP	0.89 (0.78–1.02)	0.097
CTI	0.95 (0.80–1.12)	0.543
SHR	0.96 (0.85–1.08)	0.478
METS-IR	0.78 (0.56–1.09)	0.143
TyG-BMI	0.75 (0.50–1.14)	0.180

Values are hazard ratios with 95% confidence intervals per 1-SD increase in each index. All indices were examined using the same multivariable Cox regression framework adjusted for age, sex, education, marital status, race, alcohol use, smoking, diabetes, high cholesterol, hypertension, sleep disorder, moderate physical activity, income, coronary heart disease, stroke, general health, C-reactive protein, and body mass index. Abbreviations: AIP, atherogenic index of plasma; CHG, cholesterol–high-density lipoprotein–glucose index; CI, confidence interval; CTI, cardiometabolic index; HDL-C, high-density lipoprotein cholesterol; HR, hazard ratio; METS-IR, metabolic score for insulin resistance; SHR, stress hyperglycemia ratio; TG, triglycerides; TyG, triglyceride–glucose index; UI, urinary incontinence.

Table S6. Sensitivity analysis excluding urinary incontinence events within the first 2 years

Variables	Model 1		Model 2		Model 3	
	HR (95% CI)	*P* value	HR (95% CI)	*P* value	HR (95% CI)	*P* value
**CHG (per 1 unit)**	0.66 (0.44~0.97)	0.037	0.63 (0.42~0.94)	0.024	0.55 (0.33~0.93)	0.026
**CHG (per 1 SD)**	0.89 (0.79~0.99)	0.037	0.88 (0.78~0.98)	0.024	0.85 (0.73~0.98)	0.026
**CHG Tertiles**						
T1	1.0 [Ref]		1.0 [Ref]		1.00 [Ref]	
T2	0.79 (0.61~1.04)	0.090	0.73 (0.56~0.96)	0.022	0.78 (0.56~1.07)	0.123
T3	0.76 (0.58~0.99)	0.044	0.73 (0.56~0.96)	0.026	0.68 (0.48~0.96)	0.030
P-trend		0.041		0.023		0.028

Model 1: unadjusted for any covariates.

Model 2: adjusted for age, gender, education, marital status, race.

Model 3: adjusted for age, gender, education, marital status, race, alcohol use, diabetes, smoking, high cholesterol, hypertension, sleep disorder, moderate physical activity, job income, coronary heart disease, stroke, general health, CRP, BMI.

Abbreviation: CHG, cholesterol, high-density lipoprotein, and glucose; UI, urinary incontinence; BMI, Body mass index; CRP, C-reactive protein; HR, Hazard ratio.

Table S7. Sensitivity analysis of the association between CHG and incident urinary incontinence after additional adjustment for frailty index

Variables	Model 1		Model 2		Model 3		Model 4	
	HR (95% CI)	*P* value	HR (95% CI)	*P* value	HR (95% CI)	*P* value	HR (95% CI)	*P* value
**CHG (per 1 unit)**	0.66 (0.44~0.97)	0.037	0.63 (0.42~0.94)	0.024	0.49 (0.31~0.77)	0.002	0.48 (0.31~0.74)	<0.001
**CHG (per 1 SD)**	0.89 (0.79~0.99)	0.037	0.88 (0.78~0.98)	0.024	0.82 (0.72~0.93)	0.002	0.81 (0.72~0.92)	<0.001
**CHG Tertiles**								
T1	1.0 [Ref]		1.0 [Ref]		1.0 [Ref]		1.0 [Ref]	
T2	0.79 (0.61~1.04)	0.090	0.73 (0.56~0.96)	0.022	0.68 (0.51~0.90)	0.007	0.71 (0.54~0.93)	0.012
T3	0.76 (0.58~0.99)	0.044	0.73 (0.56~0.96)	0.026	0.63 (0.47~0.85)	0.002	0.62 (0.47~0.84)	0.002
P-trend		0.041		0.023		0.002		0.001

Model 1: unadjusted for any covariates.

Model 2: adjusted for age, gender, education, marital status, race.

Model 3: adjusted for age, gender, education, marital status, race, alcohol use, smoking, diabetes, high cholesterol, hypertension, sleep disorder, moderate physical activity, job income, coronary heart disease, stroke, general health, CRP, BMI.

Model 4: additionally adjusted for frailty index.

Abbreviation: CHG, cholesterol, high-density lipoprotein, and glucose; UI, urinary incontinence; BMI, Body mass index; CRP, C-reactive protein; HR, Hazard ratio.

Table S8. Sensitivity analysis of the association between CHG and incident urinary incontinence after additional adjustment for pain, mobility impairment, and history of falls

Variables	Model 1		Model 2		Model 3		Model 4	
	HR (95% CI)	*P* value	HR (95% CI)	*P* value	HR (95% CI)	*P* value	HR (95% CI)	*P* value
**CHG (per 1 unit)**	0.66 (0.44~0.97)	0.037	0.63 (0.42~0.94)	0.024	0.49 (0.31~0.77)	0.002	0.48 (0.31~0.74)	<0.001
**CHG (per 1 SD)**	0.89 (0.79~0.99)	0.037	0.88 (0.78~0.98)	0.024	0.82 (0.72~0.93)	0.002	0.81 (0.72~0.92)	<0.001
**CHG Tertiles**								
T1	1.0 [Ref]		1.0 [Ref]		1.0 [Ref]		1.0 [Ref]	
T2	0.79 (0.61~1.04)	0.090	0.73 (0.56~0.96)	0.022	0.68 (0.51~0.90)	0.007	0.71 (0.54~0.93)	0.012
T3	0.76 (0.58~0.99)	0.044	0.73 (0.56~0.96)	0.026	0.63 (0.47~0.85)	0.002	0.62 (0.47~0.84)	0.002
P-trend		0.041		0.023		0.002		0.001

Model 1: unadjusted for any covariates.

Model 2: adjusted for age, gender, education, marital status, race.

Model 3: adjusted for age, gender, education, marital status, race, alcohol use, smoking, diabetes, high cholesterol, hypertension, sleep disorder, moderate physical activity, job income, coronary heart disease, stroke, general health, CRP, BMI.

Model 4: additionally adjusted for pain, mobility impairment, and history of falls.

Abbreviation: CHG, cholesterol, high-density lipoprotein, and glucose; UI, urinary incontinence; BMI, Body mass index; CRP, C-reactive protein; HR, Hazard ratio.

Table S9. Sensitivity analysis of the association between CHG and incident urinary incontinence after additional adjustment for antihypertensive medication use

Variables	Model 1		Model 2		Model 3		Model 4	
	HR (95% CI)	*P* value	HR (95% CI)	*P* value	HR (95% CI)	*P* value	HR (95% CI)	*P* value
**CHG (per 1 unit)**	0.66 (0.44~0.97)	0.037	0.63 (0.42~0.94)	0.024	0.49 (0.31~0.77)	0.002	0.48 (0.31~0.74)	<0.001
**CHG (per 1 SD)**	0.89 (0.79~0.99)	0.037	0.88 (0.78~0.98)	0.024	0.82 (0.72~0.93)	0.002	0.81 (0.72~0.92)	<0.001
**CHG Tertiles**								
T1	1.0 [Ref]		1.0 [Ref]		1.0 [Ref]		1.0 [Ref]	
T2	0.79 (0.61~1.04)	0.090	0.73 (0.56~0.96)	0.022	0.68 (0.51~0.90)	0.007	0.71 (0.54~0.93)	0.012
T3	0.76 (0.58~0.99)	0.044	0.73 (0.56~0.96)	0.026	0.63 (0.47~0.85)	0.002	0.62 (0.46~0.83)	0.001
P-trend		0.041		0.023		0.002		0.001

Model 1: unadjusted for any covariates.
Model 2: adjusted for age, gender, education, marital status, race.
Model 3: adjusted for age, gender, education, marital status, race, alcohol use, smoking, diabetes, high cholesterol, hypertension, sleep disorder, moderate physical activity, job income, coronary heart disease, stroke, general health, CRP, BMI.
Model 4: additionally adjusted for antihypertensive medication use
Abbreviation: CHG, cholesterol, high-density lipoprotein, and glucose; UI, urinary incontinence; BMI, Body mass index; CRP, C-reactive protein; HR, Hazard ratio.

Table S10. Sensitivity analysis of the association between CHG and incident urinary incontinence after additional adjustment for diabetes medication use

Variables	Model 1		Model 2		Model 3		Model 4	
	HR (95% CI)	*P* value	HR (95% CI)	*P* value	HR (95% CI)	*P* value	HR (95% CI)	*P* value
**CHG (per 1 unit)**	0.66 (0.44~0.97)	0.037	0.63 (0.42~0.94)	0.024	0.49 (0.31~0.77)	0.002	0.48 (0.31~0.74)	<0.001
**CHG (per 1 SD)**	0.89 (0.79~0.99)	0.037	0.88 (0.78~0.98)	0.024	0.82 (0.72~0.93)	0.002	0.81 (0.72~0.92)	<0.001
**CHG Tertiles**								
T1	1.0 [Ref]		1.0 [Ref]		1.0 [Ref]		1.0 [Ref]	
T2	0.79 (0.61~1.04)	0.090	0.73 (0.56~0.96)	0.022	0.68 (0.51~0.90)	0.007	0.71 (0.54~0.93)	0.013
T3	0.76 (0.58~0.99)	0.044	0.73 (0.56~0.96)	0.026	0.63 (0.47~0.85)	0.002	0.62 (0.47~0.84)	0.002
P-trend		0.041		0.023		0.002		0.001

Model 1: unadjusted for any covariates.

Model 2: adjusted for age, gender, education, marital status, race.

Model 3: adjusted for age, gender, education, marital status, race, alcohol use, smoking, diabetes, high cholesterol, hypertension, sleep disorder, moderate physical activity, job income, coronary heart disease, stroke, general health, CRP, BMI.

Model 4: additionally adjusted for diabetes medication use

Abbreviation: CHG, cholesterol, high-density lipoprotein, and glucose; UI, urinary incontinence; BMI, Body mass index; CRP, C-reactive protein; HR, Hazard ratio.

Table S11. Sensitivity analysis of the association between CHG and incident urinary incontinence after additional adjustment for hemoglobin

Variables	Model 1		Model 2		Model 3		Model 4	
	HR (95% CI)	*P* value	HR (95% CI)	*P* value	HR (95% CI)	*P* value	HR (95% CI)	*P* value
**CHG (per 1 unit)**	0.66 (0.44~0.97)	0.037	0.63 (0.42~0.94)	0.024	0.49 (0.31~0.77)	0.002	0.53 (0.34~0.83)	0.006
**CHG (per 1 SD)**	0.89 (0.79~0.99)	0.037	0.88 (0.78~0.98)	0.024	0.82 (0.72~0.93)	0.002	0.84 (0.74~0.95)	0.006
**CHG Tertiles**								
T1	1.0 [Ref]		1.0 [Ref]		1.0 [Ref]		1.0 [Ref]	
T2	0.79 (0.61~1.04)	0.090	0.73 (0.56~0.96)	0.022	0.68 (0.51~0.90)	0.007	0.72 (0.55~0.95)	0.019
T3	0.76 (0.58~0.99)	0.044	0.73 (0.56~0.96)	0.026	0.63 (0.47~0.85)	0.002	0.67 (0.50~0.90)	0.009
P-trend		0.041		0.023		0.002		0.007

Model 1: unadjusted for any covariates.

Model 2: adjusted for age, gender, education, marital status, race.

Model 3: adjusted for age, gender, education, marital status, race, alcohol use, smoking, diabetes, high cholesterol, hypertension, sleep disorder, moderate physical activity, job income, coronary heart disease, stroke, general health, CRP, BMI.

Model 4: additionally adjusted for hemoglobin

Abbreviation: CHG, cholesterol, high-density lipoprotein, and glucose; UI, urinary incontinence; BMI, Body mass index; CRP, C-reactive protein; HR, Hazard ratio.

Table S12. Sensitivity analysis of the association between CHG and incident urinary incontinence after additional adjustment for gait speed

Variables	Model 1		Model 2		Model 3		Model 4	
	HR (95% CI)	*P* value	HR (95% CI)	*P* value	HR (95% CI)	*P* value	HR (95% CI)	*P* value
**CHG (per 1 unit)**	0.66 (0.44~0.97)	0.037	0.63 (0.42~0.94)	0.024	0.49 (0.31~0.77)	0.002	0.50 (0.29~0.87)	0.014
**CHG (per 1 SD)**	0.89 (0.79~0.99)	0.037	0.88 (0.78~0.98)	0.024	0.82 (0.72~0.93)	0.002	0.82 (0.70~0.96)	0.014
**CHG Tertiles**								
T1	1.0 [Ref]		1.0 [Ref]		1.0 [Ref]		1.0 [Ref]	
T2	0.79 (0.61~1.04)	0.090	0.73 (0.56~0.96)	0.022	0.68 (0.51~0.90)	0.007	0.77 (0.55~1.08)	0.137
T3	0.76 (0.58~0.99)	0.044	0.73 (0.56~0.96)	0.026	0.63 (0.47~0.85)	0.002	0.59 (0.40~0.85)	0.005
P-trend		0.041		0.023		0.002		0.005

Model 1: unadjusted for any covariates.

Model 2: adjusted for age, gender, education, marital status, race.

Model 3: adjusted for age, gender, education, marital status, race, alcohol use, smoking, diabetes, high cholesterol, hypertension, sleep disorder, moderate physical activity, job income, coronary heart disease, stroke, general health, CRP, BMI.

Model 4: additionally adjusted for gait speed

Abbreviation: CHG, cholesterol, high-density lipoprotein, and glucose; UI, urinary incontinence; BMI, Body mass index; CRP, C-reactive protein; HR, Hazard ratio.

Table S13. Sensitivity analysis excluding participants with diabetes at baseline

Variables	Model 1		Model 2		Model 3	
	HR (95% CI)	*P* value	HR (95% CI)	*P* value	HR (95% CI)	*P* value
**CHG (per 1 unit)**	0.66 (0.44~0.97)	0.037	0.63 (0.42~0.94)	0.024	0.47 (0.30~0.74)	0.001
**CHG (per 1 SD)**	0.89 (0.79~0.99)	0.037	0.88 (0.78~0.98)	0.024	0.82 (0.72~0.92)	0.001
**CHG Tertiles**						
T1	1.0 [Ref]		1.0 [Ref]		1.0 [Ref]	
T2	0.79 (0.61~1.04)	0.090	0.73 (0.56~0.96)	0.022	0.64 (0.48~0.85)	0.002
T3	0.76 (0.58~0.99)	0.044	0.73 (0.56~0.96)	0.026	0.64 (0.48~0.86)	0.003
P-trend		0.041		0.023		0.002

Model 1: unadjusted for any covariates.

Model 2: adjusted for age, gender, education, marital status, race.

Model 3: adjusted for age, gender, education, marital status, race, alcohol use, smoking, high cholesterol, hypertension, sleep disorder, moderate physical activity, job income, coronary heart disease, stroke, general health, CRP, BMI.

Abbreviation: CHG, cholesterol, high-density lipoprotein, and glucose; UI, urinary incontinence; BMI, Body mass index; CRP, C-reactive protein; HR, Hazard ratio.

Table S14. Sensitivity analysis excluding participants with coronary heart disease or stroke at baseline

Variables	Model 1		Model 2		Model 3	
	HR (95% CI)	*P* value	HR (95% CI)	*P* value	HR (95% CI)	*P* value
**CHG (per 1 unit)**	0.66 (0.44~0.97)	0.037	0.63 (0.42~0.94)	0.024	0.52 (0.32~0.82)	0.005
**CHG (per 1 SD)**	0.89 (0.79~0.99)	0.037	0.88 (0.78~0.98)	0.024	0.83 (0.73~0.95)	0.005
**CHG Tertiles**						
T1	1.0 [Ref]		1.0 [Ref]		1.00 [Ref]	
T2	0.79 (0.61~1.04)	0.090	0.73 (0.56~0.96)	0.022	0.70 (0.53~0.94)	0.016
T3	0.76 (0.58~0.99)	0.044	0.73 (0.56~0.96)	0.026	0.60 (0.44~0.82)	0.001
P-trend		0.041		0.023		0.001

Model 1: unadjusted for any covariates.

Model 2: adjusted for age, gender, education, marital status, race.

Model 3: adjusted for age, gender, education, marital status, race, alcohol use, diabetes, smoking, high cholesterol, hypertension, sleep disorder, moderate physical activity, job income, general health, CRP, BMI.

Abbreviation: CHG, cholesterol, high-density lipoprotein, and glucose; UI, urinary incontinence; BMI, Body mass index; CRP, C-reactive protein; HR, Hazard ratio.

Table S15. Net benefit of the base model and the CHG-added model at selected decision thresholds

Decision threshold, %	Base model net benefit	CHG-added model net benefit	Difference in net benefit
**5**	0.0373	0.0376	0.0003
**10**	0.0170	0.0159	-0.0011
**15**	0.0063	0.0081	0.0018
**20**	0.0035	0.0057	0.0022
**25**	0.0031	0.0040	0.0008

Abbreviations: CHG, cholesterol–high-density lipoprotein–glucose index.

Note: Net benefit was estimated using decision curve analysis at selected decision thresholds. The base model included demographic, lifestyle, and clinical covariates. The CHG-added model included the base model plus CHG. Difference in net benefit was calculated as the net benefit of the CHG-added model minus that of the base model.

Table S16. Sensitivity analyses using alternative CHG cut-points for incident urinary incontinence

CHG categorization	Contrast	Model 1		Model 2		Model 3	
		HR (95% CI)	*P* value	HR (95% CI)	*P* value	HR (95% CI)	*P* value
**Continuous CHG**	Per 1-SD increase	0.89 (0.79–0.99)	0.037	0.88 (0.78–0.98)	0.024	0.82 (0.72–0.93)	0.002
**Tertiles**	T2 vs T1	0.79 (0.61-1.04)	0.09	0.73 (0.56-0.96)	0.022	0.68 (0.51-0.90)	0.007
**Tertiles**	T3 vs T1	0.76 (0.58-0.99)	0.044	0.73 (0.56-0.96)	0.026	0.63 (0.47-0.85)	0.002
Tertiles	P for trend	0.87 (0.76-0.99)	0.041	0.85 (0.74-0.98)	0.023	0.79 (0.68-0.92)	0.002
Tertiles, combined	T2–T3 vs T1	0.78 (0.62–0.98)	0.032	0.74 (0.58–0.93)	0.009	0.66 (0.52–0.85)	0.001
Median split	High vs low	0.85 (0.68–1.06)	0.141	0.84 (0.67–1.05)	0.128	0.78 (0.62–1.00)	0.048
Quartiles	Q2 vs Q1	1.01 (0.74–1.37)	0.964	0.93 (0.68–1.26)	0.620	0.85 (0.62–1.17)	0.315
Quartiles	Q3 vs Q1	0.92 (0.68–1.26)	0.609	0.85 (0.63–1.17)	0.320	0.79 (0.57–1.10)	0.157
Quartiles	Q4 vs Q1	0.77 (0.56–1.07)	0.123	0.76 (0.55–1.05)	0.096	0.64 (0.45–0.91)	0.013
Quartiles	P for trend	0.92 (0.83–1.02)	0.106	0.91 (0.83–1.01)	0.082	0.87 (0.78–0.97)	0.013
Quartiles, combined	Q2–Q4 vs Q1	0.90 (0.70–1.16)	0.415	0.85 (0.66–1.09)	0.198	0.77 (0.59–1.01)	0.056

Model 1: unadjusted for any covariates.

Model 2: adjusted for age, gender, education, marital status, race.

Model 3: adjusted for age, gender, education, marital status, race, alcohol use, smoking, diabetes, high cholesterol, hypertension, sleep disorder, moderate physical activity, job income, coronary heart disease, stroke, general health, CRP, BMI.

Abbreviation: CHG, cholesterol, high-density lipoprotein, and glucose; UI, urinary incontinence; BMI, Body mass index; CRP, C-reactive protein; HR, Hazard ratio.

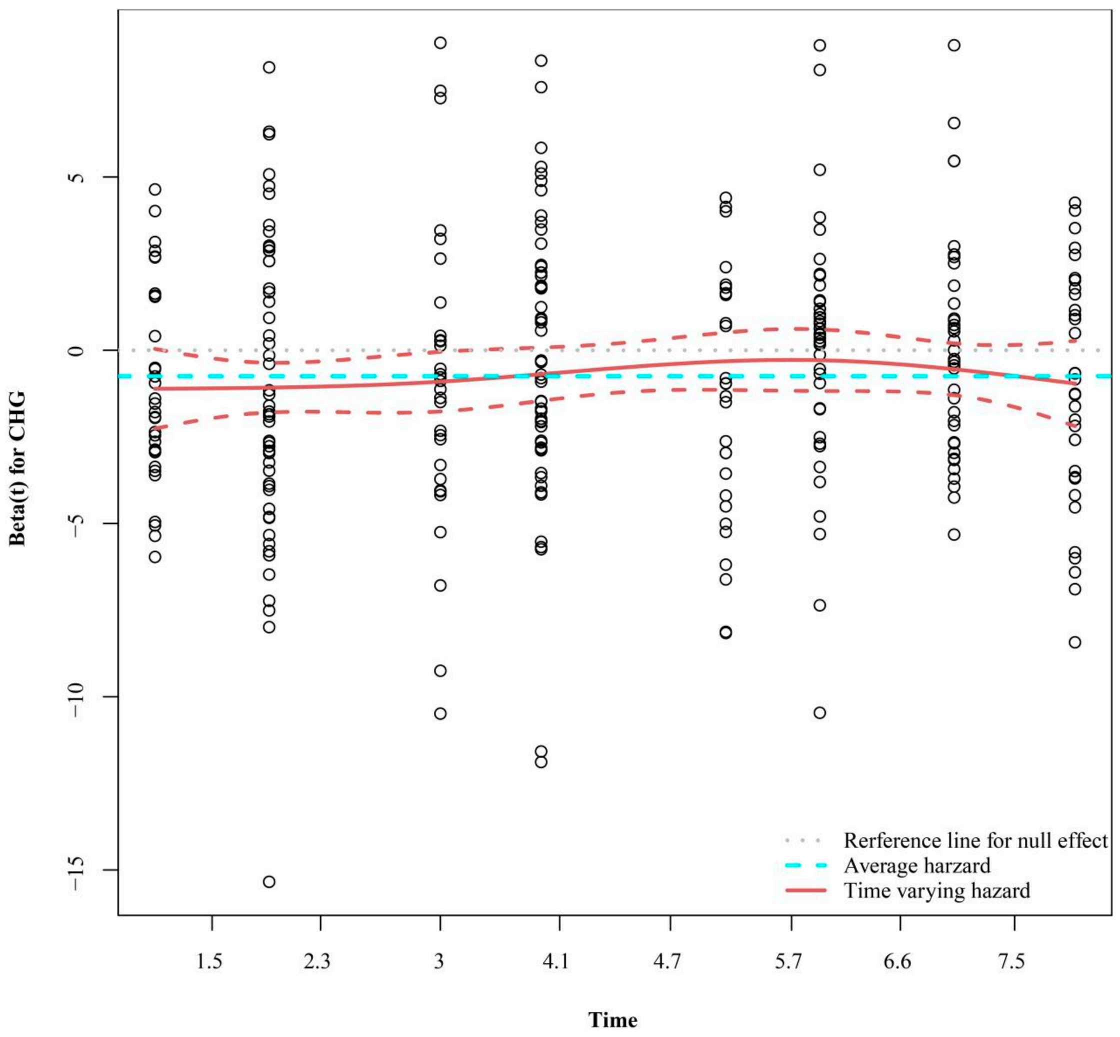

Figure S1. Assessment of the proportional hazards assumption for CHG.

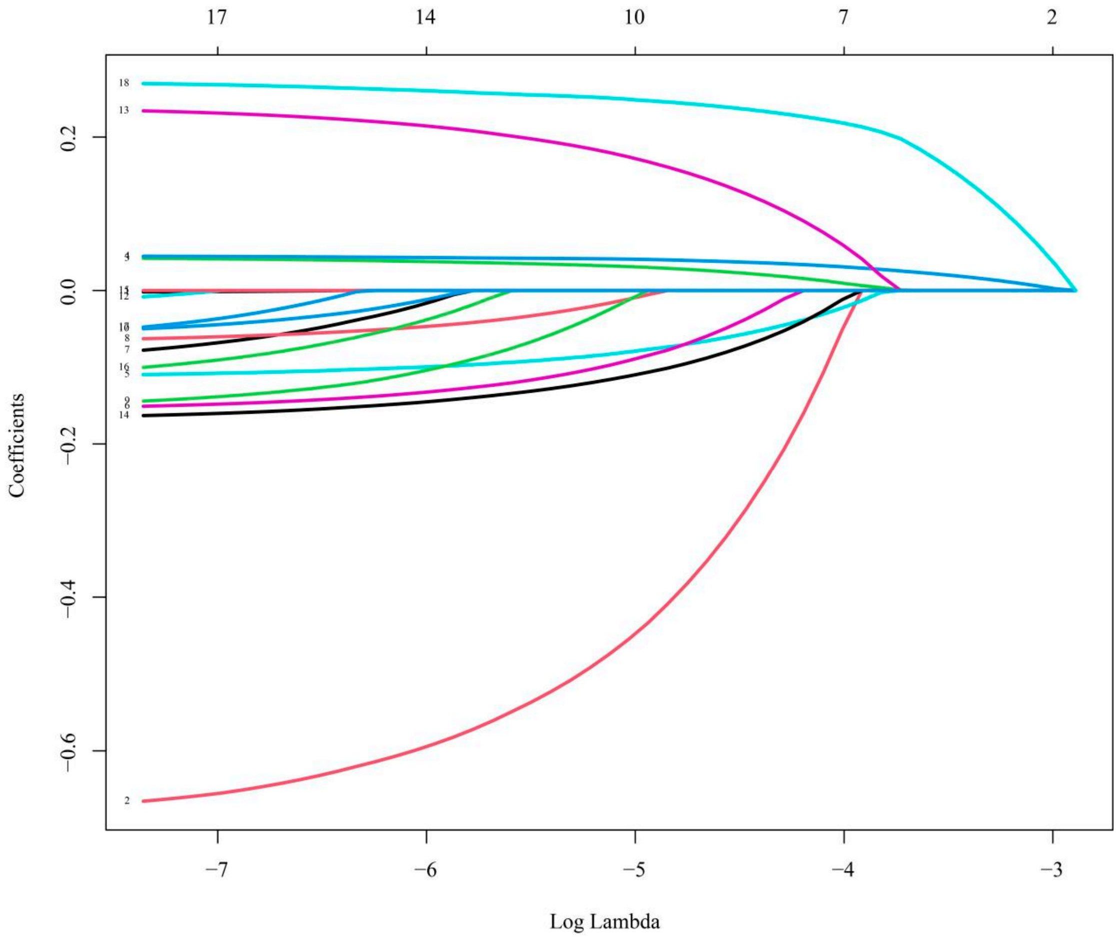

Figure S2. Coefficient profiles of candidate predictors in the LASSO regression.

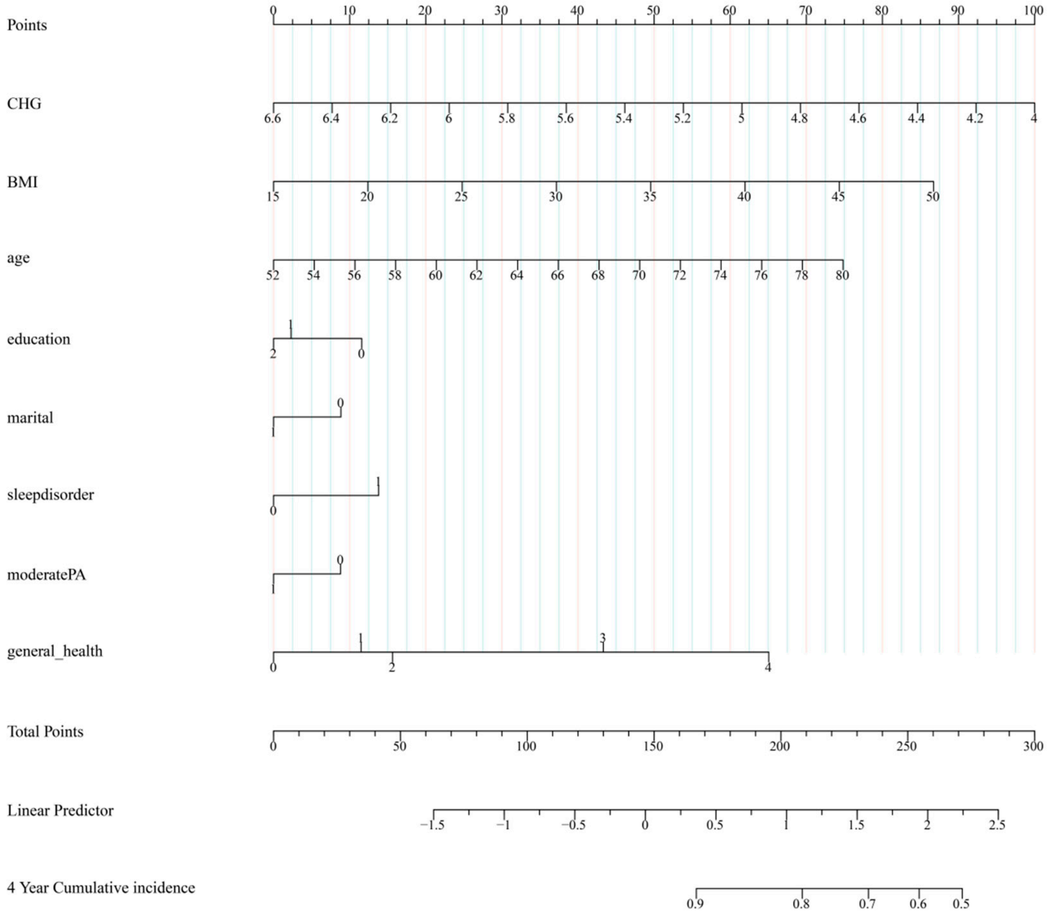

Figure S3. Nomogram for estimating 4-year urinary incontinence risk.

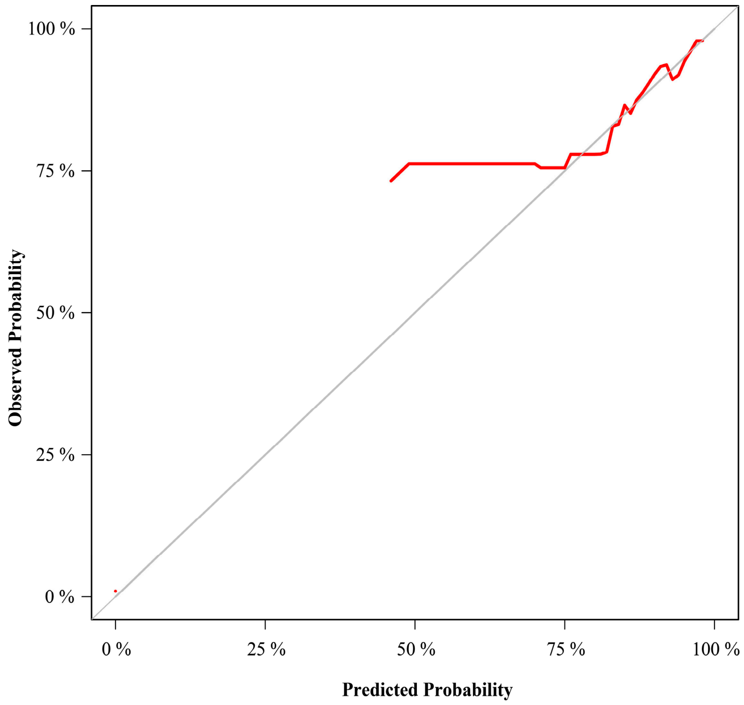

Figure S4. Calibration plot of the 4-year urinary incontinence prediction model.

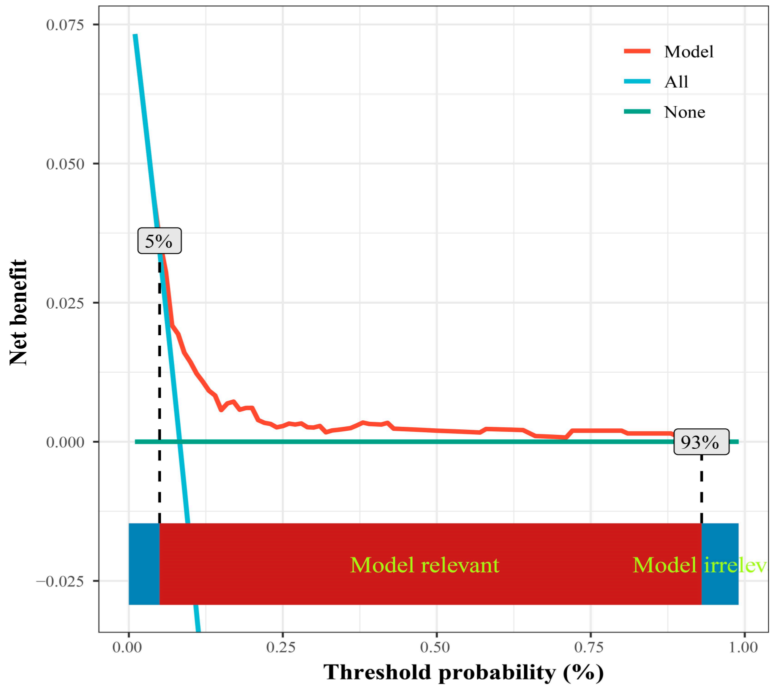

Figure S5. Decision curve analysis of the 4-year urinary incontinence prediction model.

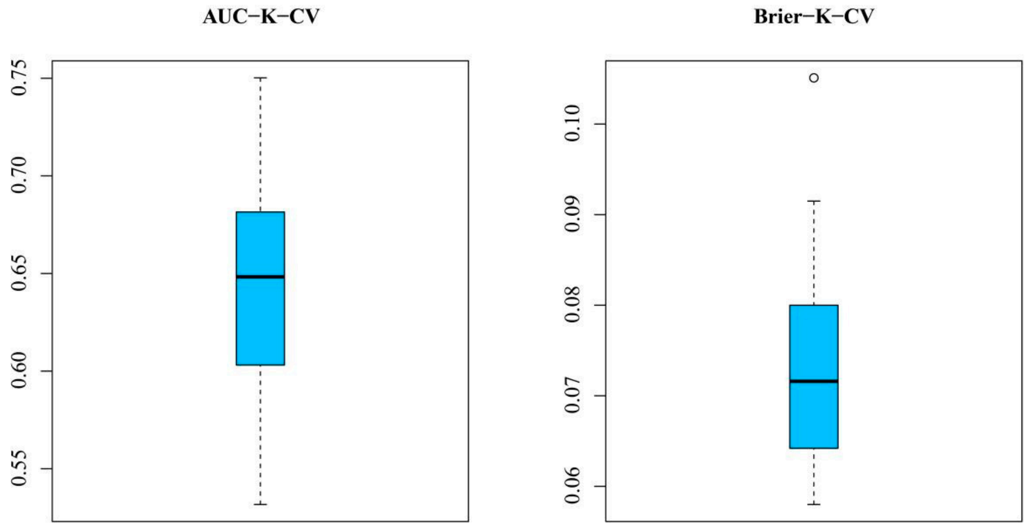

Figure S6. Internal validation performance of the 4-year urinary incontinence prediction model.
